# Microbiological Characterization of VNRX-5236, a Broad-Spectrum β-Lactamase Inhibitor for Rescue of the Orally Bioavailable Cephalosporin Ceftibuten as a Carbapenem-Sparing Agent against Strains of *Enterobacterales* Expressing Extended-Spectrum β-Lactamases and Serine Carbapenemases

**DOI:** 10.1128/AAC.00552-21

**Published:** 2021-07-16

**Authors:** Cassandra L. Chatwin, Jodie C. Hamrick, Robert E. L. Trout, Cullen L. Myers, Susan M. Cusick, William J. Weiss, Mark E. Pulse, Luigi Xerri, Christopher J. Burns, Gregory Moeck, Denis M. Daigle, Kaitlyn John, Tsuyoshi Uehara, Daniel C. Pevear

**Affiliations:** a Venatorx Pharmaceuticals, Incorporated, Malvern, Pennsylvania, USA; b UNT System College of Pharmacy, University of North Texas Health Science Center, Fort Worth, Texas, USA; c MTF Biologics, Edison, New Jersey, USA

**Keywords:** VNRX-7145, VNRX-5236 etzadroxil, ceftibuten, *Enterobacterales*, oral antibiotics

## Abstract

There is an urgent need for oral agents to combat resistant Gram-negative pathogens. Here, we describe the characterization of VNRX-5236, a broad-spectrum boronic acid β-lactamase inhibitor (BLI), and its orally bioavailable etzadroxil prodrug, VNRX-7145. VNRX-7145 is being developed in combination with ceftibuten, an oral cephalosporin, to combat strains of *Enterobacterales* expressing extended-spectrum β-lactamases (ESBLs) and serine carbapenemases. VNRX-5236 is a reversible covalent inhibitor of serine β-lactamases, with inactivation efficiencies on the order of 10^4^ M^−1^ · sec^−1^, and prolonged active site residence times (*t*_1/2_, 5 to 46 min). The spectrum of inhibition includes Ambler class A ESBLs, class C cephalosporinases, and class A and D carbapenemases (KPC and OXA-48, respectively). Rescue of ceftibuten by VNRX-5236 (fixed at 4 μg/ml) in isogenic strains of Escherichia coli expressing class A, C, or D β-lactamases demonstrated an expanded spectrum of activity relative to oral comparators, including investigational penems, sulopenem, and tebipenem. VNRX-5236 rescued ceftibuten activity in clinical isolates of *Enterobacterales* expressing ESBLs (MIC_90_, 0.25 μg/ml), KPCs (MIC_90_, 1 μg/ml), class C cephalosporinases (MIC_90_, 1 μg/ml), and OXA-48-type carbapenemases (MIC_90_, 1 μg/ml). Frequency of resistance studies demonstrated a low propensity for recovery of resistant variants at 4× the MIC of the ceftibuten/VNRX-5236 combination. *In vivo*, whereas ceftibuten alone was ineffective (50% effective dose [ED_50_], >128 mg/kg), ceftibuten/VNRX-7145 administered orally protected mice from lethal septicemia caused by Klebsiella pneumoniae producing KPC carbapenemase (ED_50_, 12.9 mg/kg). The data demonstrate potent, broad-spectrum rescue of ceftibuten activity by VNRX-5236 in clinical isolates of cephalosporin-resistant and carbapenem-resistant *Enterobacterales*.

## INTRODUCTION

The *Enterobacterales* order is composed of Gram-negative bacterial pathogens that cause frequent infections in both the community and health care settings. β-Lactam antibiotics are employed frequently to treat infections by strains of *Enterobacterales*. Indeed, β-lactams are the most commonly prescribed antibiotics, representing over 60% of prescriptions written globally, with the cephalosporin class representing nearly 50% of those prescriptions (1, 2). However, the continued expansion of resistance mechanisms in *Enterobacterales* is compromising the efficacy of β-lactam antibiotics globally (https://externalwebapps.lahey.org/Studies/).

β-Lactamases represent the primary mechanism by which most Gram-negative bacteria develop resistance to β-lactams (3, 4). These enzymes can be categorized according to amino acid sequence relatedness into four classes based on the Ambler classification system (5). Ambler class A enzymes include original spectrum β-lactamases (OSBL; which target penicillins predominantly) such as TEM-1, extended spectrum β-lactamases (ESBLs) such as CTX-M-15 and SHV-5, and carbapenemases such as KPC and GES-5. Ambler class C enzymes (PDC, AmpC, and FOX) can be encoded chromosomally or on plasmids and hydrolyze most penicillins and cephalosporins (5). Ambler class D “OXA” enzymes are a large group of beta-lactamases that include both cephalosporinases (OXA-1) and carbapenemases (e.g., OXA-23 and OXA-48). Ambler class A, C, and D enzymes all have a canonical serine active site nucleophile that attacks the β-lactam carbonyl, resulting in hydrolysis of the β-lactam moiety, thus rendering the antibiotic inert. Clinically relevant Ambler class B (metallo) enzymes use a di-zinc metal center to generate a hydroxyl nucleophile and include NDM, VIM, SPM, and IMP, among others (5). While there are more than 7,000 β-lactamases identified to date, those that are found most commonly in *Enterobacterales* are ESBLs that have specificity for most penicillins, cephalosporins, and monobactams (https://externalwebapps.lahey.org/Studies/). The CDC estimates that there were 197,400 cases of ESBL-producing *Enterobacterales* in hospitalized patients in 2017 (6). Alarmingly, in 2017 there were also an estimated 13,100 hospitalized patients with infections due to carbapenem-resistant *Enterobacterales*, including those expressing KPC and OXA-48-type carbapenemases (7).

Broad-spectrum, orally bioavailable antibiotics play a critical role in the management of human infectious diseases, in both the hospital and community settings, facilitating exit from the intensive care unit and treatment in the general ward and early discharge and/or preventing emergency department visits or hospitalization in the first place. Over 5,000,000 patients are treated in hospitals for Gram-negative infections annually in the United States alone (8). In the hospital general ward and ICU, the switch from parenteral antibiotics to oral therapy typically occurs once vital signs are stabilized and the patient is sufficiently alert. Such a treatment strategy has been shown to have a significant positive impact on overall outcomes for patients, as well as associated health care costs (9–13). The oral β-lactams (oral penicillins and cephalosporins in particular) are a key component of these therapies, including for the pediatric population. However, for indications where *Enterobacterales* strains are major causative pathogens (e.g., urinary tract infections; UTI), the future of these oral β-lactams is threatened due to the emergence and spread of β-lactamase-producing strains that inactivate these agents and that are not inhibited by the commercially available oral β-lactamase inhibitor, clavulanic acid. Further complicating matters, these β-lactamase-producing pathogens are also increasingly cross-resistant to several other classes of oral antibiotics, including quinolones and trimethoprim-sulfamethoxazole, which further limits therapeutic options (14–17). While new oral penem β-lactams are in development, these agents do not provide a solution for the ever-expanding plethora of carbapenemases found in strains of *Enterobacterales*, which is concerning considering that studies have shown that a significant percentage of carbapenem-resistant *Enterobacterales* (CRE) infections may be originating from outside the health care system in the community (18, 19). While several parenteral β-lactam/β-lactamase inhibitor (BL/BLI) combinations have been approved recently or are in late-stage development, such agents cannot address the need for flexibility in treatment of community infections or to shorten the length of stay for patients with infections initially treated in the hospital (20–25).

**FIG 1:**
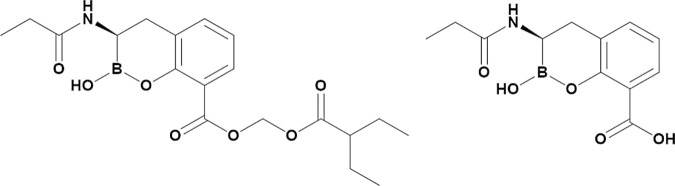
Structures of orally bioavailable prodrug VNRX-7145 (VNRX-5236 etzadroxil) (left) and active BLI, VNRX-5236 (right).

Broadly active, orally bioavailable BL/BLI combinations are a critical need to treat infections in the hospital and community. We present here comprehensive biochemical and microbiological data describing the broad-spectrum activity of a new cyclic boronate BLI, VNRX-5236 (Fig. 1) in combination with the 3rd-generation oral cephalosporin, ceftibuten (CTB). Our findings provide the biochemical basis for broad-spectrum inhibition of β-lactamases (including serine carbapenemases) by VNRX-5236 and show that addition of this next-generation BLI restores antibacterial activity of CTB against *Enterobacterales* producing clinically important serine β-lactamases, including CTX-M-, KPC-, OXA-, and class C enzymes.

## RESULTS

### Biochemical studies demonstrating selective inhibition of serine β-lactamases, including carbapenemases.

The 50% inhibition concentrations (IC_50_s) were determined for VNRX-5236 against a panel of serine β-lactamases. As shown in Table 1, VNRX-5236 had broad inhibitory activity against serine β-lactamases from Ambler A, C, and D classes, including the carbapenemases KPC-2 and OXA-48. Overall, IC_50_ values for VNRX-5236 were comparable to those for avibactam and markedly lower than those for tazobactam and clavulanic acid against KPC-2 (0.08, 0.06, 1.7, and 1.8 μM, respectively), OXA-48 (0.32, 0.55, 3.5, and 14.3 μM, respectively), and both plasmid-borne (CMY-2: 0.01, 0.007, 0.41, and >100 μM, respectively) and chromosomal (P99: 0.01, 0.02, 0.73, and >100 μM, respectively) class C enzymes. None of these inhibitors had appreciable activity against Ambler class B metallo-β-lactamases (data not shown).

**TABLE 1:** VNRX-5236 50% inhibition concentrations against serine β-lactamases^*a*^

Ambler class	β-lactamase	IC_50_ (μM) for:			
		VNRX-5236	Avibactam	Tazobactam	Clavulanic acid
A	CTX-M-15	0.02	0.003	0.001	0.04
	KPC-2	0.08	0.06	1.7	1.8
C	P99AmpC	0.01	0.02	0.73	>100
	CMY-2	0.01	0.007	0.41	>100
D	OXA-1	0.07	0.04	0.43	0.12
	OXA-48	0.32	0.55	3.5	14.3

a Substrates were nitrocefin for P99AmpC, CMY-2, OXA-1, and OXA-48; cefotaxime for CTX-M-15 and SHV-5; and imipenem for KPC-2. IC_50_ values are reported as the mean from duplicate measurements.

VNRX-5236 exhibited potent inhibition of CTX-M-15 and SHV-5 (class A ESBLs), KPC-2 (class A carbapenemase), and P99AmpC (class C cephalosporinase), with inhibitor potency (*K_i_*) values ranging from 0.01 to 0.11 μM (Table 2). Like other boronic acid-containing BLIs, inhibition of serine β-lactamases by VNRX-5236 is best described by a two-step model, in which a noncovalent complex forms, followed by a reversible covalent bond with the active site serine residue. Thus, second-order rate constants (*k_2_*/*K_i_*) for covalent bond formation were determined for the aforementioned enzymes, indicating efficient inactivation by VNRX-5236, with *k_2_*/*K_i_* on the order of 10^4^ to 10^5^ M^−1^ s^−1^ (Table 2). Dissociation rates (*k*_-2_) ranged from 2.5 to 24 × 10^−4^ s^−1^, which translated to appreciable active site residence times (*t*_1/2_) from 5 to 46 min (Table 2).

**TABLE 2:** Kinetic parameters of reversible inactivation of serine β-lactamases by VNRX-5236

Parameter	Data for:			
	KPC-2^*a*^	CTX-M-15	SHV-5	P99AmpC
*k_2_*/*K_i_* (10^4^ M^−1^s^−1^)	2.9 ± 0.07	4.8 ± 0.9	1.1 ± 0.16	6.0 ± 0.6
*k*_−2_ (10^−4^ s^−1^)	2.5 ± 0.1	4.5 ± 0.1	12.7 ± 0.07	24 ± 0.5
*t*_1/2_ (min)	46 ± 2	26 ± 0.6	6.5 ± 0.04	5 ± 0.1
*K_i_* (μM)	0.11	0.01	0.04	0.02

a KPC-2, Ambler class A Klebsiella pneumoniae carbapenemase; CTX-M-15, Ambler class A cefotaximase-Munich 15 extended-spectrum β-lactamase; SHV-5, Ambler class A sulfhydryl variable β-lactamase; P99AmpC (ACT-C189), Ambler class C chromosomal-encoded β-lactamase; *k_2_*/*K*_i_, rate of covalent bond formation; *k*_−2_, off rate; *K_i_*, inhibition constant; *t*_1/2_, half-life.

### Selection of ceftibuten as an orally bioavailable partner antibiotic.

**FIG 2:**
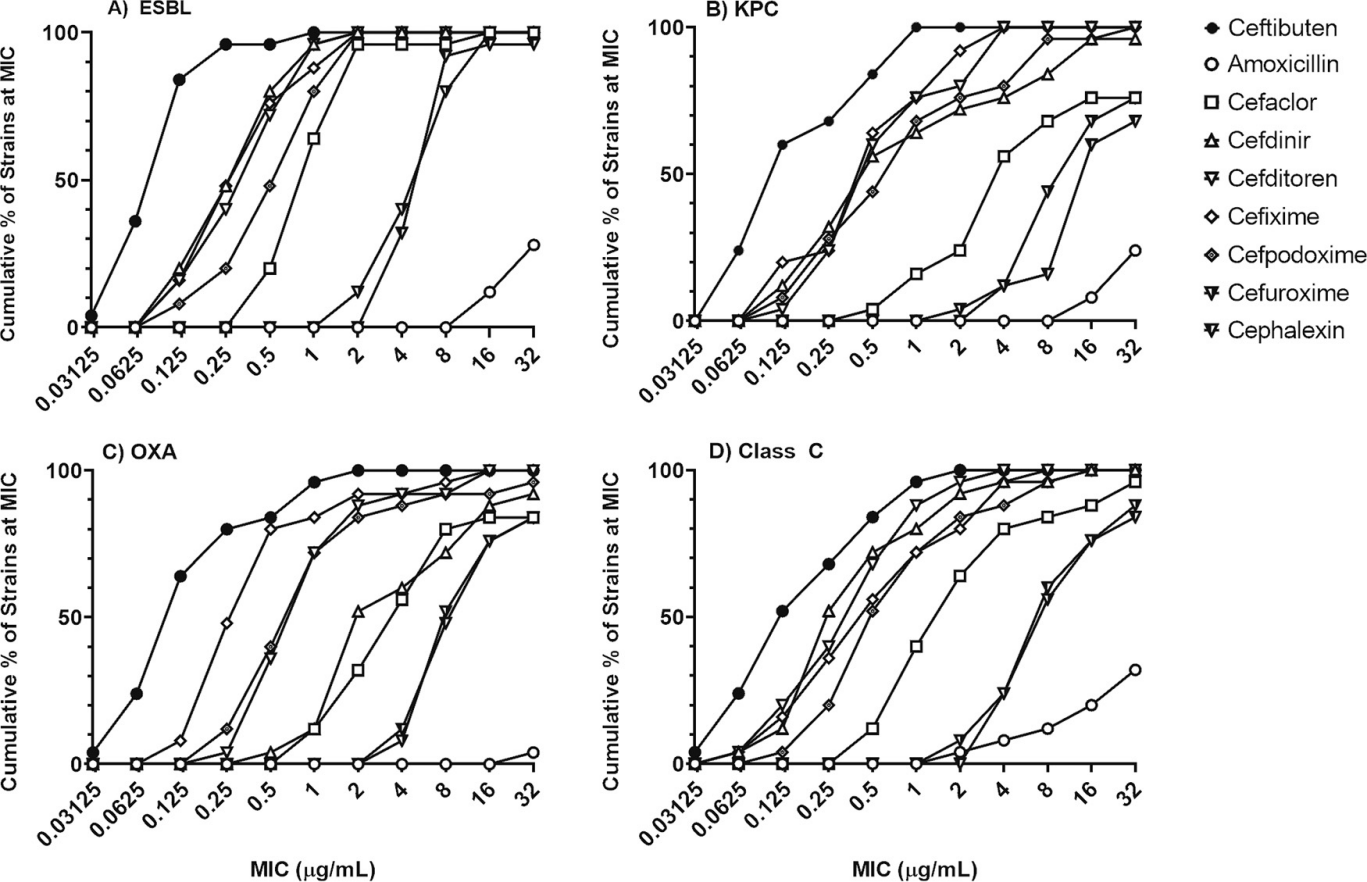
Partner β-lactam activity in combination with VNRX-5236 against strains of *Enterobacterales* expressing serine β-lactamases. All testing was conducted according to the CLSI broth microdilution method, with VNRX-5236 fixed at 4 μg/ml and partner β-lactam titrated (26). Clinical isolates tested included (A) 12 *E. coli* and 13 *K. pneumoniae*; (B) 19 *K. pneumoniae*, 1 *E. coli*, 3 *E. cloacae*, and 2 Klebsiella oxytoca; (C) 13 *K. pneumoniae* and 12 *E. coli*; (D) 7 *E. coli*, 7 *K. pneumoniae*, 6 Serratia marcescens, 3 Klebsiella aerogenes, 1 Citrobacter freundii, and 1 Proteus mirabilis.

A primary consideration for partner selection was the ability of VNRX-5236 to rescue β-lactam activity in strains of *Enterobacterales* expressing Ambler class A, C, and/or D β-lactamases, including serine carbapenemases. The activity of nine orally bioavailable β-lactam antibiotics was tested alone and in combination with VNRX-5236 (fixed at 4 μg/ml) against panels of *Enterobacterale*s strains expressing class A ESBLs, class A KPC-carbapenemases, class C cephalosporinases, or class D OXA-carbapenemases. Many strains expressed multiple enzymes. These nine potential partner antibiotics were ceftibuten, cefixime, cefditoren, cefpodoxime, cefdinir, cefaclor, cephalexin, cefuroxime, and amoxicillin. The MIC_90_ for all β-lactams tested alone against each panel of 25 strains was ≥64 μg/ml (26). As shown in Fig. 2, the combination of VNRX-5236 (fixed at 4 μg/ml) with CTB (a third-generation, orally bioavailable cephalosporin; filled circles) resulted in the lowest MICs across all sets of strains tested (MIC_50_/MIC_90_ of 0.12/1 μg/ml). VNRX-5236 also demonstrated substantial rescue of cefixime and cefditoren with an overall MIC_50_/MIC_90_ of 0.5/2 μg/ml. Amoxicillin was the weakest partner antibiotic (open circles). Coupled with its excellent oral bioavailability (∼65%), long plasma half-life (∼2.4 h), moderate human plasma protein binding (∼60%), and extensive renal excretion (∼56%), CTB was chosen as the preferred partner antibiotic (27, 28).

### Lack of stand-alone activity of VNRX-5236 and VNRX-7145 against Gram-positive and Gram-negative pathogens.

VNRX-5236 and its prodrug, VNRX-7145 (VNRX-5236 etzadroxil), lack intrinsic antibacterial activity. Neither compound demonstrated stand-alone activity against four strains of Gram-positive organisms or nine strains of Gram-negative bacilli (all MICs were ≥128 μg/ml; see Table S1 in the supplemental material).

### Impact of overexpression of key Ambler class A, B, C, and D enzymes on activity of CTB/VNRX-5236 and comparators in isogenic strains of Escherichia coli.

The impact of overexpression of key Ambler class A, B, C, and D enzymes on the activity of CTB/VNRX-5236, and by inference the breadth of inhibitory activity of the BLI, was assessed in 20 engineered E. coli strains each overproducing individual serine- or metallo-β-lactamases and compared directly to other orally available β-lactam agents, including amoxicillin-clavulanic acid, CTB/clavulanic acid, sulopenem, and tebipenem. CTB/VNRX-5236, CTB/clavulanic acid, and amoxicillin-clavulanic acid were tested with inhibitor fixed at 4 μg/ml to avoid the stand-alone activity of clavulanic acid in these strains (the modal MIC for clavulanic acid alone against these 20 strains was 32 μg/ml; data not shown). Data are presented as the fold-increase in MIC relative to the vector control strain treated with β-lactam alone. Fold increases in MIC of ≤8, highlighted in gray in Table 3, indicate the stability of the β-lactam or β-lactam/BLI combination MIC to that enzyme.

**TABLE 3:** Impact of key Ambler class A, B, C, and D enzymes on activity of CTB/VNRX-5236 and comparators in isogenic strains of E. coli^*a*^

β-lactamase	β-lactamase class	Fold increase in MIC compared to vector control for:^*b*^						
		CTB	CTB/VNRX-5236 (4)	CTB/CLA	AMX	AMX/CLA	Tebipenem	Sulopenem
TEM-10	A	4	2	4	≥128	8	4	2
CTX-M-15	A	64	0.5	1	≥128	4	4	4
GES-5	A	8	0.5	4	≥128	≥128	512	256
SHV-5	A	128	0.5	1	≥128	8	4	8
SHV-12	A	256	1	1	≥128	8	4	4
VEB-9	A	≥1,024	2	1	≥128	1	2	4
KPC-2	A	16	0.25	16	≥128	≥128	2,048	2,048
KPC-3	A	16	0.25	16	≥128	≥128	2,048	2,048
KPC-3 D179Y	A	32	2	2	4	4	8	16
PER-1	A	1,024	2	2	128	1	4	8
CMY-2	C	1,024	2	1,024	128	≥128	4	8
P99/AmpC	C	≥1,024	4	≥1,024	≥128	≥128	8	16
ACT-17	C	1,024	4	≥1,024	128	≥128	4	8
CMY-42	C	≥1,024	4	1,024	128	≥128	16	16
OXA-23	D	2	0.5	2	≥128	≥128	128	64
OXA-48	D	4	1	4	≥128	≥128	256	512
OXA-163	D	32	0.5	32	≥128	≥128	32	8
OXA-181	D	2	0.5	2	≥128	≥128	256	1,024
NDM-1	B	≥1,024	≥1,024	≥1,024	≥128	≥128	2,048	1,024

a CTB, ceftibuten; CTB/VNRX-5236 (4), ceftibuten with VNRX-5236 fixed at 4 μg/ml; CLA, clavulanic acid; AMX, amoxicillin; AMX/CLA, amoxicillin with clavulanic acid fixed at 4 μg/ml.

b MIC increases of ≤8-fold from vector control are shaded in gray and are based on modal MIC testing across 4 replicates. Vector control MICs were CTB, 1 μg/ml; CTB/VNRX-5236, 0.25 μg/ml; CTB/CLA, 1 μg/ml; AMX, 8 μg/ml; AMX/CLA, 8 μg/ml; tebipenem, 0.016 μg/ml; sulopenem, 0.06 μg/ml.

As shown in Table 3, the CTB MIC was elevated in the presence of most class A, C, and B enzymes, with the exception of TEM-10 and GES-5, which increase the CTB MIC by only 4- to 8-fold. The CTB MIC was stable for most of the class D enzymes tested. Addition of VNRX-5236 at 4 μg/ml reduced the MIC shift to ≤4-fold in all strains except for the strain expressing the Ambler class B MBL, NDM-1. Addition of clavulanic acid fixed at 4 μg/ml reduced the CTB MIC in some strains expressing ESBLs but was ineffective against class A KPC carbapenemases, class C cephalosporinases, and class D carbapenemases.

The orally bioavailable β-lactam amoxicillin was hydrolyzed extensively by all enzymes tested, with the exception of the KPC-3 D179Y variant (29). As seen with CTB, clavulanic acid had variable activity against class A ESBLs and was largely ineffective against class A KPC carbapenemases, class C cephalosporinases, and class D carbapenemases.

Tebipenem was relatively resistant to hydrolysis by class A ESBLs and class C enzymes. It was, however, hydrolyzed extensively by class A, B, and D carbapenemases. Sulopenem had an enzyme hydrolysis profile similar to that of tebipenem.

### Potentiation of CTB activity by VNRX-5236 in *Enterobacterales.*

The antibacterial activity of CTB/VNRX-5236 and comparators was assessed against a diverse panel of 100 clinical isolates of *Enterobacterales* with defined β-lactamase subtypes (Table 4). The panel does not reflect current epidemiological trends but instead highlights differences in coverage provided by CTB/VNRX-5236 relative to comparators. Antibacterial activity of CTB alone was included to ascertain the level of potentiation by the partnered BLI when tested at a fixed concentration of 4 μg/ml in all combinations except amoxicillin-clavulanic acid, where a fixed 2:1 ratio was tested (30, 31). Isolates were subdivided by molecular characterization and included 25 strains of *Enterobacterale*s expressing: (i) mixed class A ESBLs and OSBL (typically TEM-1), (ii) class A KPC-type carbapenemases, (iii) mixed class A ESBLs and OSBLs and class C cephalosporinases, and (iv) OXA-48/48-like carbapenemases.

**TABLE 4:** Activity of CTB/VNRX-5236 and comparators against *Enterobacterales* expressing class A ESBLs, class A carbapenemases (KPC), class C cephalosporinases, and class D oxacillinases^*a*^

Ambler class type	Antimicrobial agent	MIC range (μg/ml)	MIC_50_ (μg/ml)	MIC_90_ (μg/ml)
A-ESBL^*b*^	CTB	0.12 to ≥64	8	≥64
	CTB/VNRX-5236	**0.03–1**	**0.12**	**0.25**
	CTB/CLA	0.03 to ≥64	0.12	0.5
	AMC	8 to ≥64	16	32
	Sulopenem	0.03–1	0.12	0.5
	Tebipenem	≤0.016–0.12	0.03	0.06
A-KPC^*c*^	CTB	0.5 to ≥64	16	≥64
	CTB/VNRX-5236	**0.06–1**	**0.12**	**1**
	CTB/CLA	0.25 to ≥64	8	≥64
	AMC	32 to ≥256	≥64	≥256
	Sulopenem	4 to ≥64	32	≥64
	Tebipenem	8 to ≥64	≥64	≥64
C^*d*^	CTB	2 to ≥64	16	≥64
	CTB/VNRX-5236	**0.03–2**	**0.12**	**1**
	CTB/CLA	0.06 to ≥64	2	≥64
	AMC	8 to ≥256	32	≥256
	Sulopenem	0.03–8	0.06	2
	Tebipenem	≤0.016–2	0.06	0.5
D^*e*^	CTB	0.06 to ≥64	16	≥64
	CTB/VNRX-5236	**0.03–2**	**0.12**	**1**
	CTB/CLA	0.06 to ≥64	4	32
	AMC	128 to ≥256	≥256	≥256
	Sulopenem	1 to ≥64	8	16
	Tebipenem	0.5 to ≥64	4	16

a CTB, ceftibuten; CTB/VNRX-5236, ceftibuten with VNRX-5236 fixed at 4 μg/ml (boldface); CTB/CLA, ceftibuten with clavulanic acid fixed at 4 μg/ml; AMC, amoxicillin with clavulanic acid tested at a 2:1 ratio.

b Includes 12 strains of E. coli and 13 strains of K. pneumoniae.

c Includes 1 strain of E. coli, 19 strains of K. pneumoniae, 3 strains of E. cloacae, and 2 strains of Klebsiella oxytoca.

d Includes 7 strains of E. coli, 7 strains of K. pneumoniae, 3 strains of Klebsiella aerogenes, 6 strains of Serratia marcescens, 1 strain of Proteus mirabilis, and 1 strain of Citrobacter freundii.

e Includes 12 strain of E. coli and 13 strains of K. pneumoniae.

The activity of CTB alone against isolates expressing Ambler class A ESBLs and OSBLs was variable, with an MIC_50_ and MIC_90_ of 8 and ≥64 μg/ml, respectively (Table 4). This result is consistent with the variable impact of class A enzyme expression on CTB activity in the isogenic strain panel (Table 3). VNRX-5236 rescued CTB activity against the clinical strains, with an MIC_50_ and MIC_90_ of 0.12 and 0.25 μg/ml. As these strains express enzymes that are variably susceptible to inhibition by clavulanic acid, the CTB MIC_90_ was reduced from ≥64 with CTB alone to 0.5 μg/ml for CTB/clavulanic acid. Amoxicillin-clavulanic acid had elevated MICs across these strains (MIC_90_ of 32 μg/ml). The investigational oral penems demonstrated reasonably uniform activity against these strains, with MIC_90_s of 0.5 (sulopenem) and 0.06 (tebipenem) μg/ml, respectively.

CTB alone demonstrated very high MIC_50_ and MIC_90_ values of 16 and ≥64 μg/ml, respectively, against strains expressing KPC-type carbapenemases. VNRX-5236 rescued CTB activity against these strains, with an MIC_50_/MIC_90_ of 0.12 and 1 μg/ml, respectively, for the combination. Clavulanic acid was ineffective at rescuing CTB or amoxicillin in these strains. In addition, the MIC_50_/MIC_90_ for sulopenem (32/≥64 μg/ml) and tebipenem (≥64/≥64 μg/ml) demonstrated very poor activity of these agents against KPC-expressing isolates.

CTB alone demonstrated elevated MIC_50_/MIC_90_ values of 16/≥64 μg/ml against strains expressing class C cephalosporinases. These values were reduced to 0.12/1 μg/ml, respectively, in the presence of VNRX-5236. The strains had elevated CTB/clavulanic acid and AMC MICs (MIC_90_, ≥64 and ≥256 μg/ml, respectively), highlighting the weak protection afforded by clavulanic acid. The oral penems demonstrated good activity against these strains, with the MIC_90_ for sulopenem and tebipenem being 2 and 0.5 μg/ml, respectively.

CTB demonstrated weak activity against strains expressing class D enzymes, with MIC_50_/MIC_90_ values of 16 and ≥64 μg/ml, respectively. VNRX-5236 reduced the CTB MIC_50_/MIC_90_ values to 0.12 and 1 μg/ml, respectively. CTB/clavulanic acid, sulopenem and tebipenem all demonstrated weak activity against these strains, with MIC_50_ of 4 to 8 μg/ml and MIC_90_ of 16 to 32 μg/ml. Amoxicillin-clavulanic acid did not demonstrate significant activity against these strains (MIC range, 128 to ≥256 μg/ml).

### Assessment of bactericidal activity of CTB/VNRX-5236.

**FIG 3:**
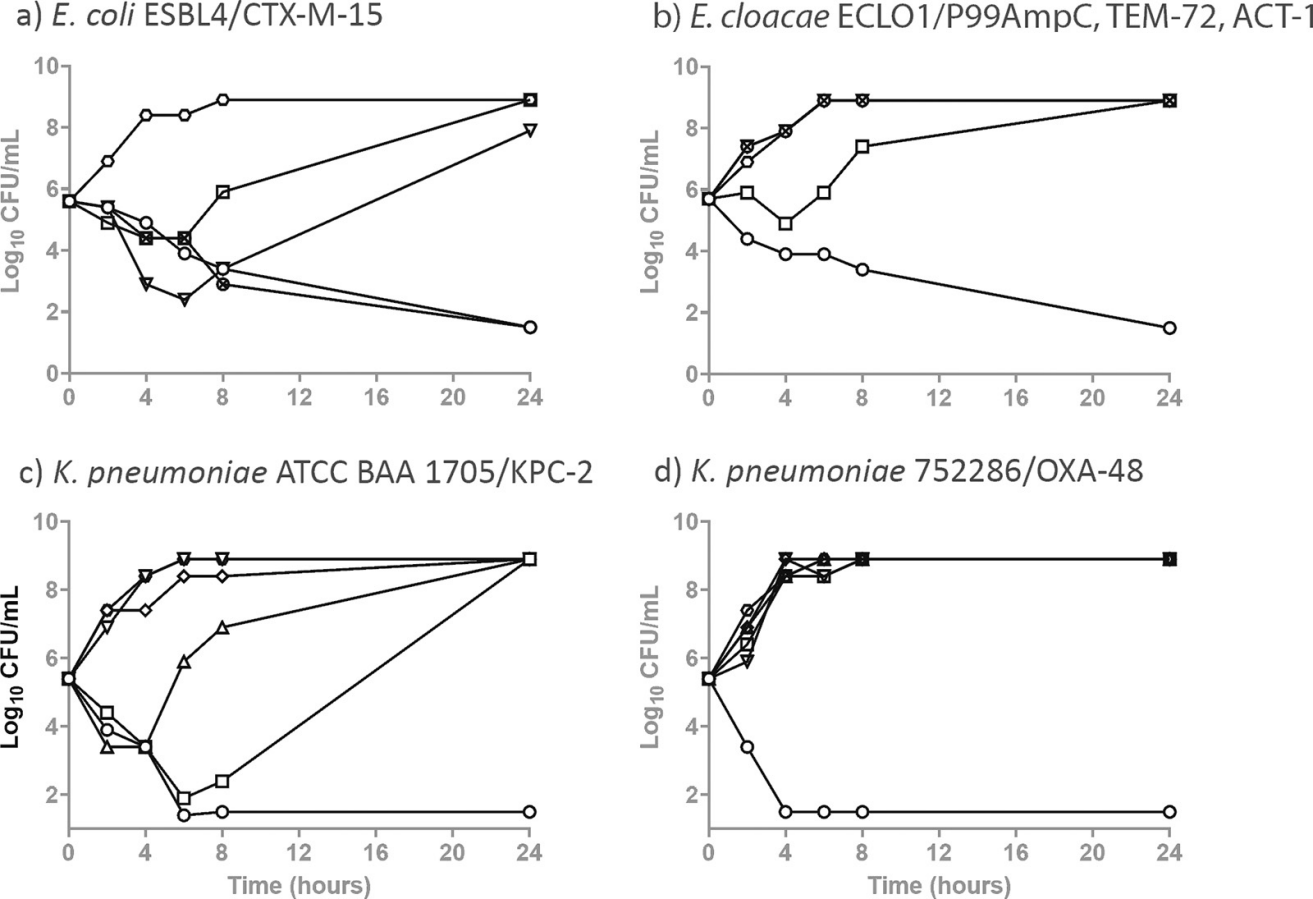
Time-kill assay of CTB/VNRX-5236 against strains of *Enterbacterales* with defined resistance mechanisms. Hexagon, growth control; square, CTB alone at 8 μg/ml; open circle, CTB at 2 μg/ml with VNRX-5236 fixed at 4 μg/ml; × inside a circle, CTB at 2 μg/ml with clavulanic acid fixed at 4 μg/ml; upside-down triangle, amoxicillin at 8 μg/ml with clavulanic acid at 4 μg/ml (a 2:1 ratio); triangle, tebipenem at 2 μg/ml; diamond, sulopenem at 2 μg/ml.

To assess the ability of VNRX-5236 to restore the bactericidal activity of CTB against resistant isolates, 24-h time-kill assays were conducted. Shown in Table 5 are the modal MIC and minimum bactericidal concentration (MBC) values for antibacterial agents against these four bacterial strains. Shaded boxes represent data for the compound/pathogen combination shown in Fig. 3. VNRX-5236 restored CTB activity in all strains tested, reducing the CTB-alone MIC from a range of 16 to 128 μg/ml to 0.12 to 1 μg/ml. The investigational penems demonstrated potent activity against strains expressing ESBLs or class C cephalosporinases, but as expected, their activity was compromised in the presence of carbapenemases such as KPC-2 and OXA-48.

**TABLE 5:** MICs of antimicrobial agents against strains utilized in time-kill studies^*a*^

Antimicrobial agent	Organism, strain (β-lactamase content)							
	E. coli ESBL4 (CTX-M-15)		E. cloacae ECL01 (TEM-72, ACT-1, P99AmpC)		K. pneumoniae ATCC BAA-1705 (KPC-2)		K. pneumoniae 752286 (OXA-48)	
	MIC (μg/ml)	MBC (μg/ml)	MIC (μg/ml)	MBC (μg/ml)	MIC (μg/ml)	MBC (μg/ml)	MIC (μg/ml)	MBC (μg/ml)
CTB	32	>32	64	>32	16	16	128	≥128
CTB-CLA	0.12	1	≥256	≥256	8	16	16	16
CTB/VNRX-5236	0.12	0.12	0.25	1	0.12	0.25	0.25	0.25
AMC	16	16	128	≥128	≥256	≥256	≥256	≥256
Sulopenem	0.12	0.12	1	1	32	32	16	>32
Tebipenem	≤0.06	0.06	0.25	0.25	32	32	16	>32
Levofloxacin	32	32	0.12	0.12	64	≥64	32	>32

a MICs represent modal broth microdilution values from 5 independent determinations. MBCs are the lowest concentrations of compound tested in the time-kill experiment at 24 h that resulted in a ≥3-log_10_ CFU/ml reduction in bacterial counts from input. Shaded boxes represent data for the compound/pathogen combination shown in Fig. 3.

Both CTB/VNRX-5236 and CTB/clavulanic acid demonstrated bactericidal activity against E. coli expressing CTX-M-15 (Fig. 3a). Amoxicillin-clavulanic acid and CTB alone or with clavulanic acid demonstrated initial killing, but the strains grew to control levels by 24 h (Fig. 3a). Both tebipenem and sulopenem demonstrated bactericidal activity against this strain at drug concentrations at or near their respective MICs (see Table 5). CTB/VNRX-5236 likewise demonstrated potent bactericidal activity against Enterobacter cloacae ECL01 expressing the class A ESBL TEM-72 and 2 class C enzymes (P99AmpC = ACT-C189 and ACT-1) (Fig. 3b), as did the penems and levofloxacin (Table 5). All other antibacterial agents failed in this time-kill assay, consistent with their elevated MICs. Results against the carbapenemase-expressing strains of K. pneumoniae are shown in Fig. 3c and d. Both strains had elevated MICs to all antimicrobial agents (8 to ≥256 μg/ml), with the exception of CTB/VNRX-5236 (0.12 to 0.25 μg/ml). CTB/VNRX-5236 demonstrated bactericidal activity against these 2 strains, with MBCs within 1 dilution of their respective MIC.

### Frequency of resistance (FoR).

Experiments were conducted to assess the FoR for the CTB/VNRX-5236 combination at 4× MIC. As shown in Table 6, no resistant colonies were obtained after 48 h of incubation. The FoR for the combination (defined as CFU in the presence of CTB/VNRX-5236 divided by total CFU plated) was <4.3 × 10^−10^ for all strains tested.

**TABLE 6:** Frequency of resistance to CTB/VNRX-5236

Strain	ID	β-lactamase content	Total CFU plated	CFU CTB/VNRX-5236	FoR^*a*^
E. coli	ATCC 25922	None	3.1 × 10^9^	0	<3.2 × 10^–10^
K. pneumoniae	ATCC BAA-1705	KPC-2	2.3 × 10^9^	0	<4.3 × 10^–10^
E. cloacae	ECL01	AmpC, TEM-72, ACT-1	3.6 × 10^9^	0	<2.8 × 10^–10^
E. coli	ESBL4	CTX-M-15, TEM-1	1.2 × 10^10^	0	<8.3 × 10^–11^
K. pneumoniae	ATCC 700603	SHV-18, TEM-1	2.7 × 10^9^	0	<3.7 × 10^–10^
E. coli	VER	OXA-48	2.5 × 10^9^	0^*b*^	<4 × 10^–10^

a FoR, frequency of resistance, calculated as CFU of (CTB/VNRX-5236)/total CFU plated. Selective plates contained CTB at 4× the MIC with VNRX-5236 fixed at 4 μg/ml.

b A single colony grew on these plates but upon subculturing and broth microdilution testing the colony was found to have parental (naive) CTB/VNRX-5236 MIC.

### Impact of pH and human urine on activity of CTB/VNRX-5236.

With urinary tract infections as the targeted indication for CTB/VNRX-5236, the activity of the combination over a range of relevant pH values in the presence of pooled human urine (100%) was assessed (32). Testing in urine at pH 6.0 produced MIC results equal to or within 1 to 3 doubling dilutions compared to standard broth microdilution for CTB and CTB/VNRX-5236 (Table 7). Testing in urine at pH 7.2 produced MIC results equal to or 1 to 2 doubling dilutions lower than standard broth microdilution for CTB and CTB/VNRX-5236.

**TABLE 7:** Impact of pH and human urine on activity of CTB/VNRX-5236^*a*^

Strain	ID	Median or modal MIC (μg/ml)					
		MHII broth		Human urine, pH 6		Human urine, pH 7.2	
		CTB	CTB/VNRX-5236	CTB	CTB/VNRX-5236	CTB	CTB/VNRX-5236
E. coli	ATCC 25922	0.25	0.12	0.25	0.12	0.12	≤0.06
K. pneumoniae	ATCC 700603	0.5	0.25	1	0.5	0.5	0.12
K. pneumoniae	ATCC BAA-1705	8	0.25	2	0.5	2	≤0.06
K. pneumoniae	1434760	>128	0.5	>128	1	128	0.5
E. coli	1480076	128	0.25	32	0.5	32	0.12
K. pneumoniae	1266420	32	0.12	64	1	16	≤0.06

a CTB, ceftibuten; CTB/VNRX-5236, ceftibuten with VNRX-5236 fixed at 4 μg/ml; MHII broth, cation-adjusted Mueller-Hinton broth.

### *In vivo* efficacy of CTB/VNRX-5236 and CTB/VNRX-7145 in murine septicemia model.

The ability of VNRX-5236 (dosed subcutaneously) or VNRX-7145 (VNRX-5236 etzadroxil dosed orally) to rescue CTB activity (dosed identically to the BLI) was assessed in a lethal murine septicemia model as described elsewhere (33). Briefly, mice were administered a lethal intraperitoneal inoculum of ceftibuten-resistant K. pneumoniae UNT-023 expressing KPC, TEM-1, and SHV-11 (CTB MIC, 64 μg/ml; CTB/VNRX-5236 MIC, 0.06 μg/ml). One hour later, antimicrobial agents were administered as a single dose by the route indicated. The experiment was conducted with groups of 5 mice and run twice in order to calculate median effective dose (ED_50_) values with 95% confidence limits by probit analysis. As shown in Table 8, administration of CTB alone was ineffective at protecting mice from lethal disease (ED_50_, >128 mg/kg). In contrast, CTB/VNRX-5236 etzadroxil dosed orally or CTB/VNRX-5236 dosed subcutaneously demonstrated robust activity with respective ED_50_ values of 12.9 and 13.5 mg/kg. The results demonstrate potent rescue of CTB by VNRX-5236 and VNRX-7145 in this model.

**TABLE 8:** Efficacy of CTB/VNRX-5236 and CTB/VNRX-7145 against K. pneumoniae UNT-023 expressing KPC, TEM-1, and SHV-11^*a*^

Antimicrobial agent	MIC (μg/ml)	Route of administration	ED_50_ (mg/kg)	95% confidence limit
CTB alone	64	PO	>128	NA
CTB/VNRX-7145	ND	PO/PO	12.9	9.8–17.2
CTB/VNRX-5236	0.25	SC/SC	13.5	9.1–22.8

a ^ ^NA, not applicable due to lack of protection; ND, not determined, as VNRX-7145 (VNRX-5236 etzadroxil) has no demonstrable BLI activity; PO, *per os* (by mouth); SC, subcutaneous; ED_50_, 50% effective dose.

### High selectivity and lack of mammalian cytotoxicity.

The selectivity and specificity of VNRX-5236 and its etzadroxil prodrug were evaluated in the DrugMatrixScreen panel of pharmacological targets at Eurofins/Panlabs (34). Binding, enzymatic, and uptake assays representing a wide range of cellular and subcellular target classes were performed. At the screening concentration of 100 μM for VNRX-5236, two modest hits were reported—56% inhibition of protein serine/threonine phosphatase, PPP3CA (calcineurin PP2B), and 77% inhibition of protein tyrosine kinase Fyn. The *in vivo* significance of these *in vitro* activities is not known. VNRX-7145 (VNRX-5236 etzadroxil) was tested at a concentration of 10 μM, with no notable off-target findings reported against any of the 129 *in vitro* assays. VNRX-5236 and VNRX-7145 (VNRX-5236 etzadroxil) were also found to be noncytotoxic to dividing HeLa, MRC-5, and 3T3 cell cultures up to the highest concentration tested (1 mg/ml and 128 μg/ml, respectively).

## DISCUSSION

In spite of recent approvals of intravenous BL/BLI combinations for clinical use, no orally bioavailable BL/BLI combination has been approved in several decades to address the plethora of new β-lactamases. Two orally bioavailable investigational penem prodrugs have recently completed clinical phase 3 studies, with mixed results. Sulopenem etzadroxil failed to achieve noninferiority relative to ertapenem injection in patients with complicated urinary tract infections (cUTI; SURE-2 trial) or complicated intra-abdominal infections (cIAI; SURE-3 trial) (35–37). Tebipenem pivoxil hydrobromide recently demonstrated noninferiority relative to intravenous (i.v.) ertapenem in cUTI, including pyelonephritis (ADAPT-PO trial) (38). While the oral penems generally escape hydrolysis by Ambler class A ESBLs and class C cephalosporinases, they remain vulnerable to inactivation by class A and D serine carbapenemases. As such, unprotected oral penems lack utility as a step-down therapy in hospitalized patients with infections caused by carbapenemase-producing Gram-negative bacteria. Moreover, there are potential antimicrobial stewardship concerns in that widespread use of unprotected penems could further increase carbapenem resistance, which is already considered a major and growing issue worldwide (39–43).

Ceftibuten/VNRX-5236 demonstrates potent antibacterial activity against *Enterobacterales* producing Ambler class A serine β-lactamases, class C cephalosporinases, and class A and D carbapenemases (e.g., KPC and OXA-48-type, respectively) and competes directly with other early-stage BL/BLI combinations (44, 45). Such coverage is of critical importance in light of the ever-expanding contribution of these enzymes to β-lactam resistance globally (46, 47). Indeed, the previous 2 decades have seen an explosive, global expansion of ESBLs in strains of *Enterobacterales* in both the hospital and community settings (48). Such strains are considered a “critical priority” by the World Health Organization, along with strains of *Enterobacterales* expressing carbapenemases (49). Since their first reporting in 1996 in North Carolina, KPC-expressing strains of *Enterobacterales* have become endemic in many parts of the world, including the northeastern United States and Puerto Rico, much of South America, eastern China, parts of southern Europe, and Israel (50, 51). Likewise, since its discovery in Turkey in 2003, the OXA-48 carbapenemase has been identified in many parts of the world, including in the Mediterranean region and European countries (52, 53). The rapid expansion of OXA-48 in strains of *Enterobacterales*, particularly in Europe, compromises the utility of approved oral β-lactams, as well as the investigational penems discussed above (53).

The selection of ceftibuten as the β-lactam partner to VNRX-5236 was driven by multiple considerations, including (i) potency in combination with VNRX-5236 against strains of *Enterobacterales*, (ii) excellent oral pharmacokinetics, (iii) modest protein binding, (iv) significant excretion in urine (optimal for UTIs), and (v) carbapenem sparing. The currently approved dose of ceftibuten, 400 mg orally once daily, is insufficient to support plasma exposures needed to cover at least 90% of these pathogens (54). As such, extensive pharmacokinetic/pharmacodynamic (PK/PD) assessments are ongoing, both *in vitro* and *in vivo*, as well as a stand-alone phase 1 clinical trial, to select a suitable dose and dosing regimen of ceftibuten in the combination and confirm its tolerability in human volunteers (55).

VNRX-7145 (VNRX-5236 etzadroxil), the prodrug of VNRX-5236, demonstrated high oral bioavailability in rats, dogs, and monkeys (R. Trout, A. Zulli, E. Mesaros, R. W. Jackson, S. Boyd, B. Liu, J. Hamrick, D. Daigle, C. Chatwin, K. John, L. McLaughlin, S. M. Cusick, D. C. Pevear, G. Moeck, L. Xerri, and C. J. Burns, submitted for publication; 56). Importantly, levels of circulating VNRX-7145 were exceedingly low at all PK time points in these species, consistent with rapid and extensive hydrolysis of the ester prodrug *in vivo* (Trout et al., submitted; 56).

VNRX-5236 potently inhibited problematic β-lactamases found commonly in *Enterobacterales*, including KPC, CTX-M-15, P99AmpC, CMY-2, OXA-1, and OXA-48. Accordingly, in isogenic E. coli strains individually expressing these important β-lactamases, VNRX-5236 reduced the ceftibuten MIC to within 4-fold of the vector control in all strains, with the exception of the MBL NDM-1. This level of rescue far exceeded that observed when clavulanic acid was paired with ceftibuten or amoxicillin. While the penems were less susceptible than cephalosporins or amino penicillins to hydrolysis by ESBLs and class C enzymes, tebipenem and sulopenem MICs were nonetheless increased against most β-lactamase-producing isogenic strains compared to the vector control. Importantly, both investigational penems were hydrolyzed extensively by KPC and OXA carbapenemases.

VNRX-5236 lacks intrinsic antibacterial activity, differentiating this BLI from those of the diazabicyclooctane (DBO) class, which includes avibactam, as well as the investigational BLIs zidebactam, durlobactam, ETX0282, and nacubactam (45, 57–63). It will be important to monitor whether the penicillin binding protein-inhibitory activity of the DBOs increases their propensity to generate resistance against organisms in which the DBO BLI is the primary contributor to antibacterial activity.

In clinical *Enterobacterales* isolates, CTB/VNRX-5236 demonstrated potent, broad-spectrum activity against strains expressing any Ambler class of serine β-lactamase. Time-kill assays indicated that CTB/VNRX-5236 remained bactericidal over 24 h against representative strains from each Ambler serine β-lactamase class, while frequency of resistance studies revealed a very low propensity for selection of resistant variants across a range of β-lactamase-expressing strains of *Enterobacterales*. Proof of concept studies in the murine septicemia model provided strong evidence of *in vivo* efficacy for ceftibuten in combination with VNRX-7145 (VNRX-5236 etzadroxil) dosed orally and VNRX-5236 dosed subcutaneously. Indeed, previously reported studies also demonstrated potent inhibition of strains of *Enterobacterales* in the neutropenic mouse thigh and ascending urinary tract infection models (64–66).

The limitations of this study are noted. First, we have not conducted prevalence-based surveillance studies to support MIC_50_ and MIC_90_ determinations globally or by geography. Those studies are just now getting under way. Nevertheless, the predominant resistance mechanism in strains of *Enterobacterales* in the United States are ESBLs, which account for about 24% of all *Enterobacterales* infections (67). As these strains will almost certainly represent the MIC_90_ for CTB/VNRX-5236, we anticipate the MIC_90_ value for the combination to be linked to this resistance mechanism. Second, a limited number of representative β-lactamases were characterized biochemically and in bacterial cells. Our focus initially was to assess activity against the most prevalent enzymes found in strains of E. coli and K. pneumoniae. Finally, this study has not considered the role of non-β-lactamase-mediated resistance mechanisms (porin downregulation, efflux upregulation, penicillin-binding protein [PBP] target mutations) on the activity of the CTB/VNRX-5236 combination. Further expansion of this testing is ongoing. The data support further development of this first-in-class, broad-spectrum, oral BLI in combination with ceftibuten.

## MATERIALS AND METHODS

### Test substances.

VNRX-5236 (lot no. RT00097-126) was synthesized at Venatorx Pharmaceuticals. Cefpodoxime (catalog [cat.] no. J66225, lot no. P16D031) was purchased from Alfa Aesar (Tewksbury, MA). Cefditoren sodium (cat. no. 104146-53-4, lot no. E1S17V11231) was purchased from BOC Sciences (Shirley, NY). Ceftibuten hydrate (lot no. RCHX17002) was purchased from Covalent Laboratories (Telangana, India). Tebipenem (cat. no. HY-A0076, lot no. 13639) was purchased from MedChemExpress (Monmouth Junction, NJ). Cephalexin (cat. no. 150585, lot no. QR12161) was purchased from MP Biomedicals (Solon, OH). Cefuroxime sodium (cat. no. 56238-63-2, lot no. 33047-32385) was purchased from Research Products International (Mount Prospect, IL). Amoxicillin (cat. no. A8523, lot no. 084M4819V), potassium clavulanate (cat. no. 33454, lot no. SZBC146XV), cefaclor (cat. no. C6895, lot no. 103M4810V), cefdinir (cat. no. C7118, lot no. 093M4817V), and levofloxacin (cat. no. 40922, lot no. BCBW2333) were purchased from Sigma-Aldrich (St. Louis, MO). Sulopenem (cat. no. 6337/10, lot no. 1A/205439) was purchased from Tocris (Minneapolis, MN).

Stock solutions (10 mg/ml) of cefixime, cefpodoxime, ceftibuten, sulopenem, tebipenem, and VNRX-5236 were prepared in dimethyl sulfoxide (DMSO). Then, 10-mg/ml stock solutions of amoxicillin, cefdinir, cefditoren, cephalexin, and clavulanic acid were prepared in 0.1 M phosphate buffer, pH 6. A 10 mg/ml stock solution of cefaclor was prepared in water, and a 5 mg/ml stock solution of levofloxacin was prepared in ethanol. These stocks were used to prepare the final test concentrations for antibacterial assays. Weights of the salts and purity of the antimicrobial agents were taken into account when preparing the stock solutions when applicable.

For biochemical analyses, VNRX-5236 (lot no. EFM00077-053) was synthesized at Venatorx Pharmaceuticals, Inc. Cephalothin (lot no. 106M4780V) and cefotaxime (lot no. 094M4726V) were purchased from Sigma (Atlanta, GA); imipenem (lot no. R038R0) was from United States Pharmacopeia (Rockville, MD); nitrocefin (batch no. N005-09) was from TOKU-E (Bellingham, WA). Stock solutions (10 mM) for cephalothin and imipenem were prepared in phosphate-buffered saline (PBS), while cefotaxime and nitrocefin (15 mM) stocks were prepared in dimethyl sulfoxide (DMSO, D128-500; Fisher Chemical, Fairlawn, NJ). Working solutions were subsequently prepared in assay buffer, according to the final concentrations required for assay. Stock solutions (10 mM) of β-lactamase inhibitors (VNRX-5236, avibactam, tazobactam, and clavulanic acid) were prepared in DMSO and used to prepare working solutions in assay buffer according to the final assay concentrations required for the assay.

### Antibacterial activity of CTB/VNRX-5236 in reference isolates and susceptibility testing methodology.

The *in vitro* activity of CTB/VNRX-5236 was measured by broth microdilution as the activity of CTB in the presence of VNRX-5236 at a fixed concentration of 4 μg/ml. The rationale for choosing 4 μg/ml of VNRX-5236 for *in vitro* testing is based on *in vitro* broth microdilution assessments across a range of fixed concentrations of VNRX-5236 and correlation of *in vitro* results with antibacterial activity in the neutropenic mouse thigh infection model (64, 66). Against 1,066 clinical urinary tract infection isolates resistant to both amoxicillin-clavulanic acid and levofloxacin, the MIC_90_ of CTB was reduced from >32 μg/ml when tested alone to 2 μg/ml at a fixed concentration of 4 μg/ml of VNRX-5236 (J. A. Karlowsky, M. A. Hackel, and D. F. Sahm, submitted for publication; 68). The MIC distribution of CTB with VNRX-5236 fixed at 4 μg/ml against CTB nonsusceptible *Enterobacterales* from multiple challenge sets of clinical isolates resembles the MIC distribution of CTB against CTB-susceptible isolates. The overlapping MIC distributions for CTB against CTB-susceptible strains and CTB/VNRX-5236 against CTB-nonsusceptible strains (with VNRX-5236 fixed at 4 μg/ml) reflect the complete or nearly complete rescue of the activity of CTB by VNRX-5236 against target organisms. Importantly, humanized dosing of CTB and matching VNRX-5236 profiles in the neutropenic murine thigh infection model reduced the bacterial burden of all isolates of *Enterobacterales* with CTB/VNRX-5236 MIC values of 2 μg/ml or below by 0 to 1 log_10_ at 24 h compared to the burden at the outset of therapy (64, 66). These results are consistent with the MIC values for CTB derived in the presence of 4 μg/ml of VNRX-5236.

### Expression plasmid construction.

Plasmid DNA, PCR product purification, and gel extractions were performed using Wizard Plus SV miniprep and SV gel and PCR extraction kits (Promega). NdeI, BamHI, and XhoI restriction enzymes, T4 DNA ligase, and E. coli BL21(DE3) competent cells were purchased from New England Biolabs. All oligonucleotide primers for PCR amplification were purchased from Integrated DNA Technologies. All PCRs were performed with Phusion high-fidelity DNA polymerase and cloning performed in E. coli DH5α subcloning efficiency chemically competent cells (Thermo Fisher). The pET9a (StrateGene) expression clones were made using PCR amplification products from molecularly characterized clinical isolates carrying the desired β-lactamase gene and cloned NdeI to BamHI into pET9a in all cases. All β-lactamases were cloned with signal peptide encoding sequences. All transformants were verified by PCR amplification, restriction endonuclease mapping, and DNA sequencing. Confirmed expression plasmids were isolated with plasmid miniprep kits (Promega) and used to transform the expression cell line E. coli BL21(DE3) or E. coli JM109(DE3).

### β-lactamase purification.

For CMY-2 and OXA-1, a 50 ml preculture of E. coli BL21(DE3) cells containing the pET9a expression vector for the individual β-lactamases was grown at 37°C overnight in lysogeny broth (LB) medium supplemented with 50 μg/ml kanamycin. Common to all β-lactamase purifications, bacterial cells were lysed by three consecutive passes through a chilled French pressure cell at 18,500 lb/in^2^ and clarified by centrifugation at 10,000 × *g* for 30 min at 4°C. The β-lactamase activity was monitored using nitrocefin at 100 μM, and purity was examined using 10% SDS-PAGE with Coomassie brilliant blue staining. Purified proteins exhibiting >95% purity with SDS-PAGE were quantified with a Pierce BCA protein assay kit and bovine serum albumin as the standard (Thermo Fisher), concentrated to a working range of 1 to 5 mg/ml and frozen at –80°C in buffer containing 10% glycerol. Column purifications were performed using an AKTA fast protein liquid chromatograph (FPLC; GE Healthcare). The purification schemes were generally similar, with enzyme-specific differences described below.

CTX-M-15 was obtained from E. coli BL21(DE3) carrying plasmid pET-CTX-M-15, grown in 2 liters of MagicMedia autoinduction medium (Invitrogen) containing 50 μg/ml kanamycin (Sigma) for 24 h at 23°C. Cells were harvested at an optical density at 600 nm (OD_600_) of 2.2 by centrifugation at 7,500 × *g* at 4°C and resuspended in 60 ml of 10 mM HEPES-NaOH (pH 7.0) supplemented with 0.5 mM EDTA. The lysate was diluted 5-fold with cold 50 mM sodium acetate (pH 4.8) and incubated overnight at 4°C. The extract was clarified by centrifugation at 14,500 × *g* at 4°C, filtered through an Amicon nitrogen concentrator with a 10-kDa cutoff filter to a volume of 50 ml, and loaded onto a HiTrap CaptoS column preequilibrated in 50 mM sodium acetate (pH 4.8). Protein was eluted by a linear gradient of 50 mM sodium acetate (pH 4.8) supplemented with 500 mM NaCl. Fractions containing active CTX-M-15 were pooled and concentrated, and the buffer was exchanged for 20 mM HEPES-NaOH (pH 7.2), 150 mM NaCl, and 10% glycerol using Amicon Ultra-15 centrifugal concentrators. CTX-M-15 was further purified using a Superdex 200 gel filtration column.

OXA-48 was obtained from E. coli BL21(DE3) carrying plasmid pET-OXA-48, grown in 2 liters of MagicMedia autoinduction medium (Invitrogen) with kanamycin as previously described for 24 h at 23°C (20). Cells were harvested at an OD_600_ of 3 by centrifugation at 7,500 × *g* at 4°C, resuspended in 60 ml of 20 mM triethanolamine buffer (pH 5.5), and then purified as described for CTX-M-15, with the exception that the final buffer contained 10 mM NaHCO_3_ to keep the critical active site lysine residue carbamylated (69).

KPC-2 was obtained from E. coli BL21(DE3) carrying plasmid pET-KPC-2, grown in 3 liters of MagicMedia autoinduction medium (Invitrogen) with kanamycin as previously described for 24 h at 23°C (20). Cells were harvested at an OD_600_ of 3.3 by centrifugation at 7,500 × *g* at 4°C and resuspended in 70 ml of 20 mM MES-NaOH (pH 5.5). The extract was clarified by centrifugation at 14,500 × *g* at 4°C, filtered through an Amicon nitrogen concentrator by use of 10-kDa cutoff filters to a volume of 50 ml, and loaded onto a HiTrap CaptoS column preequilibrated in 20 mM MES-NaOH (pH 5.5). Protein was eluted by a linear gradient of 20 mM MES-NaOH (pH 5.5) supplemented with 500 mM NaCl. KPC-2 active fractions were pooled and concentrated, and buffer was exchanged in 20 mM HEPES-NaOH (pH 7.3) and 150 mM NaCl using Amicon Ultra-15 centrifugal concentrators. KPC-2 was further purified using gel filtration chromatography with a Superdex 200 column.

P99AmpC was purified directly from the Enterobacter cloacae P99 clinical isolate after a sequence of the β-lactamase-encoding gene had been verified by DNA sequencing of PCR amplified product. To produce P99AmpC, Enterobacter cloacae P99 cells were grown in LB in the presence of a sub-MIC (0.016 μg/ml) of imipenem to induce maximal expression of the enzyme. Cells were harvested at an OD_600_ of 2.4 by centrifugation at 7,500 × *g* at 4°C, resuspended in 50 ml of 20 mM MES-NaOH (pH 5.5), and otherwise purified in a similar manner to KPC-2 described above.

SHV-5 was obtained from E. coli BL21(DE3)/pLysS carrying plasmid pET-SHV-5, grown at 25°C in 2 liters of super broth supplemented with kanamycin and chloramphenicol to an OD_600_ of 0.5, when IPTG (isopropyl-β-d-thiogalactopyranoside) was added to 0.05 mM final, and induction proceeded for 6 h. Cells were harvested by centrifugation at 5,500 g for 15 min at 4°C. The cell pellet was resuspended in 60 ml of 10 mM HEPES-NaOH (pH 7.5), and cells were lysed using a French press. The lysate was diluted 5-fold in 50 mM sodium acetate (pH 5.0) and kept at 4°C overnight. The extract was clarified by centrifugation at 14,500 × *g* at 4°C, filtered through an Amicon nitrogen concentrator by use of 10-kDa cutoff filters to a volume of 50 ml, and loaded onto a HiTrap CaptoS column equilibrated with 50 mM sodium acetate (pH 5.0). SHV-5 was eluted by a linear gradient of 50 mM sodium acetate (pH 5.0) supplemented with 500 mM NaCl. Active fractions were pooled and concentrated, and buffer was exchanged in 20 mM HEPES-NaOH (pH 7.3), 150 mM NaCl, and 10% glycerol using Amicon Ultra-15 centrifugal concentrators. Finally, the SHV-5 sample was further purified by chromatography over a Superdex 200 gel filtration column.

### Half maximal inhibitory concentration (IC_50_) determinations.

To determine IC_50_ values, VNRX-5236 was serially diluted 3-fold in PBS in 96-well microtiter plates to obtain assay concentrations ranging from 10 μM to 0.00001 μM (1 μM to 0.000001 μM with P99AmpC). An equal volume of enzyme was added (assay concentrations, 0.6 nM P99AmpC, 21 nM CTX-M-15, 3 nM KPC-2, 100 nM SHV-5), and the mixtures were preincubated for 15 min at 37°C. For AmpC, nitrocefin was then added to obtain a final concentration of 100 μM, and the absorbance at a wavelength (λ) of 486 nm (representing hydrolysis of the nitrocefin β-lactam ring) was monitored immediately using a BioTek Powerwave XS2 microplate reader. In similar fashion, using UV-transparent 96-well microtiter plates, hydrolysis of imipenem (100 μM) by KPC-2 was monitored at λ 300 nm, while cefotaxime (100 μM) hydrolysis by CTX-M-15 and SHV-5 was monitored at λ 300 nm. Assays were performed in duplicate, and the percentage inhibition relative to reaction mixtures containing no inhibitor were determined for each concentration of inhibitor. The concentration of inhibitor required to reduce the initial rate of hydrolysis by 50% (IC_50_) was determined based on the residual β-lactamase activity.

### Measurement of the onset of inhibition (*k_2_*/*K_i_*).

VNRX-5236 binds to the active site of serine β-lactamases and reacts with the catalytic serine nucleophile to form a reversible covalent bond. This interaction is described by a 2-step model for reversible inhibition (1) shown in the scheme below, where *E* refers to enzyme and *I* to inhibitor, *EI* is the noncovalent enzyme:inhibitor complex, and EI* is the covalent enzyme:inhibitor complex.

$$E+I\overset{k_{1}}{\underset{k_{-1}}{⇄}}EI\overset{k_{2}}{\underset{k_{-2}}{⇄}}EI^{*}$$

The onset of inhibition or rate of covalent complex formation (*k_2_*/*K_i_*) was determined under steady-state conditions by monitoring the progress of β-lactam hydrolysis by the respective enzymes in the presence of increasing concentrations of VNRX-5236. Assays (200 μl final volume) were performed in PBS in triplicate using 96-well microtiter plates. Cephalothin was used as the substrate for P99AmpC and CTX-M-15 (50 μM for P99AmpC; 75 μM for CTX-M-15), and reactions were initiated by the addition of enzyme (0.2 nM P99AmpC; 3 nM CTX-M-15). The decrease in the absorbance at λ 260 nm was recorded continuously on a BioTek Powerwave XS2 microplate reader. The concentrations of VNRX-5236 tested were 20 μM, 10 μM, 5 μM, 2.5 μM, 1.25 μM, 0.625 μM, 0.313 μM, 0.156 μM, 0.0781 μM, 0.0391 μM, 0.0195 μM, and 0 μM. SHV-5 reactions were initiated with 40 nM enzyme, using cefotaxime (100 μM) as the substrate. The concentrations of VNRX-5236 were as above, and the reaction progress was monitored continuously by measuring the reduction in absorbance at λ 260 nm. For KPC-2, imipenem (75 μM) was used as the substrate, and reactions were initiated with 3 nM enzyme. Reaction progress was monitored by measuring the reduction in absorbance at λ 300 nm in a continuous fashion. The concentrations of VNRX-5236 tested were 10 μM, 5 μM, 2.5 μM, 1.25 μM, 0.625 μM, 0.313 μM, 0.156 μM, 0.0781 μM, 0.0391 μM, 0.0195 μM, 0.0098 μM, and 0 μM. Time courses were fit to equation 1 to obtain the observed rate constant, *k*_obs_, at each inhibitor concentration, and *k*_2_/*K*_i_ was subsequently determined by fitting plots of inhibitor concentration versus *k*_obs_ to equation 2, which includes a correction for substrate concentration and affinity.

(1) $$A_{i}=A_{0}+v_{s}t+\left(v_{0}-v_{s}\right)[\frac{1-e^{-k_{\mathrm{obs}}t}}{k_{\mathrm{obs}}}]$$

(2) $$k_{\mathrm{obs}}=k_{-2}+\frac{k_{2}}{K_{i}}[\frac{\left[I\right]}{1+\frac{\left[S\right]}{K_{m}}}]$$

### Off rate (*k*_off_; *k*_−2_) and half-life of active site occupancy (*t*_1/2_) determination.

Off rates for VNRX-5236 with the various β-lactamases were determined by jump dilution experiments performed in triplicate. The enzyme and inhibitor were incubated at room temperature for 10 min. Enzyme:inhibitor complexes were then diluted 800-fold into reaction buffer (50 mM HEPES, pH 7.0, 0.1 mg/ml bovine serum albumin [BSA]), and 20 μl of the diluted reaction was immediately added to 180 μl of 110 μM nitrocefin in a 96-well microtiter plate, and the absorbance at λ 486 nm measured continuously on a BioTek Powerwave XS2 microplate reader. The resulting progress curves were fit to a single exponential, from which *k*_off_ was derived. The half-life was determined from equation 3.

(3) $$t_{1/2}=\frac{0.693}{k_{\mathrm{off}}}$$

### Determination of *K_i_*.

Plots of the inhibitor concentration versus inverse the initial velocity were fit to a linear equation, from which *K_i_* was derived (equation 4). The observed *k_i_* was corrected for substrate concentration and affinity by:

(4) $$K_{i}=\frac{K_{i\mathrm{observed}}}{1+\frac{\left[S\right]}{K_{m}}}$$

[*S*] and *K_m_* correspond to the substrate concentration and Michaelis Menten constant, respectively.

### Antibacterial activity.

The *in vitro* antibacterial activity of β-lactams alone or in combination with BLIs was determined in cation-adjusted Mueller-Hinton broth (CAMHB) microdilution assays according to Clinical and Laboratory Standards Institute (CLSI) recommendations (30). Antibacterial potentiation by VNRX-5236 was assayed by fixing the BLI at 4 μg/ml, while clavulanic acid was either fixed at 4 μg/ml for testing in combination with CTB or tested at an amoxicillin:clavulanic acid ratio of 2:1 (30). Inocula for broth microdilution assays were prepared using the broth culture method. The β-lactams were 2-fold serially diluted in CAMHB, with final concentrations generally ranging from 0.06 to 128 μg/ml when tested alone and from 0.016 to 32 μg/ml when tested in combination with their respective BLI. The MICs reported are modal values from 3 to 5 independent replicates performed on separate days and by separate investigators.

### Quality control (QC) ranges.

In February 2021, broth microdilution QC ranges and QC organisms for CTB and for routine testing of the CTB/VNRX-5236 combination were adopted by the Clinical and Laboratory Standards Institute (CLSI) Antimicrobial Susceptibility Testing (AST) Subcommittee. Approved QC organisms and ranges were as follows: E. coli NCTC 13353 expressing CTX-M-15 (CTB range, 16 to 64 μg/ml; CTB/VNRX-5236 range, 0.03/4 to 0.25/4 μg/ml), K. pneumoniae ATCC BAA-1705 expressing KPC-2, SHV, and TEM (4 to 32 μg/ml; 0.12/4 to 0.5/4 μg/ml), and K. pneumoniae ATCC BAA-2814 expressing KPC-3, SHV-11, and TEM (8 to 32 μg/ml; 0.5/4 to 2/4 μg/ml). These QC ranges and strains will be published in a future edition of CLSI M100.

***In vitro* antibacterial time-kill assay.** Bactericidal activity was assessed using time-kill assays according to standard CLSI methods (70). Time-kill assays were performed in 14 ml glass tubes or deep-well storage plates with a bacterial inoculum in CAMHB of 5 × 10^5^ CFU/ml. The tubes/plates were incubated at 37°C with shaking at 200 rpm, and aliquots were drawn at six time points (0, 2, 4, 6, 8, and 24 h) to conduct 0.5 log_10_ dilutions in CAMHB in 96-well plates that were incubated overnight at 37°C and used to ascertain viable bacterial counts. The lowest quantifiable amount by this method is 2 log_10_ CFU/ml.

**Spontaneous frequency of resistance.** Bacteria (2.3 × 10^9^ to 1.2 × 10^10^ CFU) were plated on cation-adjusted Mueller-Hinton agar plates containing CTB at 4× the MIC and VNRX-5236 fixed at 4 μg/ml. Plates were incubated for 48 h at 37°C, and surviving colonies were enumerated. Frequency of resistance (FoR) is defined as CFU in the presence of CTB/VNRX-5236 divided by CFU plated.

**Mammalian cell cytotoxicity.** Cytotoxicity evaluations were conducted on actively dividing cells to maximize assay sensitivity. Procedurally, 180 μl of cells were seeded in cell-culture-treated 96-well plates (Celltreat, Pepperell, MA) at a density of 2 × 10^4^ to 3.5 × 10^4^ cells/ml and incubated overnight at 37°C in a humidified, 5% CO_2_ atmosphere. The next day, compound was diluted in PBS with 6% DMSO in a 0.5 log_10_ dilution scheme at 10 times the final compound concentration array. Compound titrations (in 20 μl volume) were added to each well of the cell culture plates to affect a final 10-fold dilution ranging from 0.315 to 1,000 μg/ml for VNRX-5236, VNRX-7145 (VNRX-5236 etzadroxil), and ceftazidime. The cytotoxic agent chlorohexidine was assayed as a positive control at compound concentrations ranging from 0.31 to 100 μg/ml. The final DMSO concentration in all assays was 0.6%.

After an additional 72 h of incubation (when cells reached 90 to 100% confluence) Cell Titer 96 AQ solution was added at 20 μl per well, and plates were incubated an additional 2 to 4 h at 37°C and 5% CO_2_. The optical density of the wells was read on a Biotek Cytation 3 plate reader at 490 nm. Background signal (wells receiving Cell Titer reagent but containing no cells) was subtracted from all wells on the plate. The percent growth versus log drug concentration was plotted, and the concentrations at which 50% of the cells survived compared to growth control (CC_50_) were calculated using a sigmoidal 4 parameter curve fit (GraphPad Prism). Experiments were run in triplicate with data from all three trials included in the data analysis.

**Impact of pH and human urine on activity of CTB/VNRX-5236.** Studies were conducted by IHMA, Schaumberg, IL. Pooled human urine (100%) was used for panel preparation (BIOIVT, product code 1801844; lot BRH1464632; expiry date, 2020-03-31) and adjusted to pH 6.0 and pH 7.2. MICs were determined by broth macrodilution methodology (30, 31). Bacterial inocula were prepared in saline at approximately 1 × 10^6^ CFU/ml by dilution of a McFarland 0.5 in saline. Antibacterial panels containing 100 μl of antibacterial solution were inoculated with the adjusted inocula using 95-prong inoculator sets. Test panels were read visually after incubation following CLSI guidelines for each organism. MIC values corresponded to the first well with no visible growth.

### Selectivity testing.

The selectivity of VNRX-5236 was assessed in the DrugMatrixScreen at Eurofins Discovery Services. This screen encompasses 123 mammalian receptor and enzyme classes (and a single prokaryotic target, a β-lactamase) that can be used to assess the potential off-target activities of development candidates (34).

### Data availability.

The raw data for graphs and tables presented in this paper can be found in Tables S2 to S9.

## References

[1] Klein EY, Van Boeckel TP, Martinez EM, Pant S, Gandra S, Levin SA, Goossens H, Laxminarayan R. 2018. Global increase and geographic convergence in antibiotic consumption between 2000 and 2015. Proc Natl Acad Sci U S A 115:E3463–E3470. doi:10.1073/pnas.1717295115. 29581252. 29581252 PMC5899442

[2] Bush K, Bradford PA. 2016. β-Lactams and β-lactamase inhibitors: an overview. Cold Spring Harb Perspect Med 6:a025247. doi:10.1073/pnas.1717295115. 27329032 PMC4968164

[3] Bush K. 2010. Alarming β-lactamase-mediated resistance in multidrug-resistant Enterobacteriaceae. Curr Opin Microbiol 13:558–564. doi:10.1016/j.mib.2010.09.006. 20920882

[4] Livermore DM. 2009. Has the era of untreatable infections arrived? J Antimicrob Chemother 64 Suppl 1:i29–i36. doi:10.1093/jac/dkp255. 19675016

[5] Ambler RP. 1980. The structure of beta-lactamases. Philos Trans R Soc Lond B Biol Sci 289:321–331. doi:10.1098/rstb.1980.0049. 6109327

[6] CDC. 2019. ESBL-producing Enterobacterales in Healthcare Settings. https://www.cdc.gov/hai/organisms/ESBL.html.

[7] CDC. 2019. Carbapenem-resistant Enterobacterales (CRE) https://www.cdc.gov/hai/organisms/cre.html.

[8] 2013. Arlington Medical Resources’ (AMR’s) Hospital Antibiotic Market Guide. Arlington Medical Resources, Inc.

[9] Dagan R, Syrogiannopoulos G, Ashkenazi S, Engelhard D, Einhorn M, Gatzola-Karavelli M, Shalit I, Amir J. 1994. Parenteral-oral switch in the management of paediatric pneumonia. Drugs 47(Suppl 3):43–51. doi:10.2165/00003495-199400473-00008. 7518766

[10] Cunha BA. 2001. Intravenous to oral antibiotic switch therapy. Drugs Today (Barc) 37:311–319. doi:10.1358/dot.2001.37.5.627953. 12768219

[11] Mertz D, Koller M, Haller P, Lampert ML, Plagge H, Hug B, Koch G, Battegay M, Flückiger U, Bassetti S. 2009. Outcomes of early switching from intravenous to oral antibiotics on medical wards. J Antimicrob Chemother 64:188–199. doi:10.1093/jac/dkp131. 19401304 PMC2692500

[12] Barlow GD, Nathwani D. 2000. Sequential antibiotic therapy. Curr Opin Infect Dis 13:599–607. doi:10.1097/00001432-200012000-00004. 11964828

[13] Lelekis M, Gould IM. 2001. Sequential antibiotic therapy for cost containment in the hospital setting: why not? J Hosp Infect 48:249–257. doi:10.1053/jhin.2001.1006. 11461124

[14] McDanel J, Schweizer M, Crabb V, Nelson R, Samore M, Khader K, Blevins AE, Diekema D, Chiang HY, Nair R, Perencevich E. 2017. Incidence of extended-spectrum β-lactamase (ESBL)-producing Escherichia coli and Klebsiella Infections in the United States: a systematic literature review. Infect Control Hosp Epidemiol 38:1209–1215. doi:10.1017/ice.2017.156. 28758612

[15] Thaden JT, Fowler VG, Sexton DJ, Anderson DJ. 2016. Increasing incidence of extended-spectrum β-lactamase-producing Escherichia coli in community hospitals throughout the southeastern United States. Infect Control Hosp Epidemiol 37:49–54. doi:10.1017/ice.2015.239. 26458226 PMC4748740

[16] Boix-Palop L, Xercavins M, Badía C, Obradors M, Riera M, Freixas N, Pérez J, Rodríguez-Carballeira M, Garau J, Calbo E. 2017. Emerging extended-spectrum β-lactamase-producing Klebsiella pneumoniae causing community-onset urinary tract infections: a case-control-control study. Int J Antimicrob Agents 50:197–202. doi:10.1016/j.ijantimicag.2017.03.009. 28552471

[17] Mazzariol A, Bazaj A, Cornaglia G. 2017. Multi-drug-resistant Gram-negative bacteria causing urinary tract infections: a review. J Chemother 29:2–9. doi:10.1080/1120009X.2017.1380395. 29271736

[18] Cotroneo N, Rubio A, Critchley IA, Pillar C, Pucci MJ. 2020. *In vitro* and *in vivo* characterization of tebipenem, an oral carbapenem. Antimicrob Agents Chemother 64:e02240-19. doi:10.1128/AAC.02240-19. 32423950 PMC7526814

[19] Zilberberg MD, Nathanson BH, Sulham K, Fan W, Shorr AF. 2017. Carbapenem resistance, inappropriate empiric treatment and outcomes among patients hospitalized with Enterobacteriaceae urinary tract infection, pneumonia and sepsis. BMC Infect Dis 17:279. doi:10.1186/s12879-017-2383-z. 28415969 PMC5393012

[20] Hamrick JC, Docquier JD, Uehara T, Myers CL, Six DA, Chatwin CL, John KJ, Vernacchio SF, Cusick SM, Trout REL, Pozzi C, De Luca F, Benvenuti M, Mangani S, Liu B, Jackson RW, Moeck G, Xerri L, Burns CJ, Pevear DC, Daigle DM. 2019. VNRX-5133 (taniborbactam), a broad-spectrum inhibitor of serine- and metallo-β-lactamases, restores activity of cefepime in *Enterobacterales* and Pseudomonas aeruginosa. Antimicrob Agents Chemother 64:e01963-19. doi:10.1128/AAC.01963-19. PMC7038240 31871094

[21] Liu B, Trout REL, Chu GH, McGarry D, Jackson RW, Hamrick JC, Daigle DM, Cusick SM, Pozzi C, De Luca F, Benvenuti M, Mangani S, Docquier JD, Weiss WJ, Pevear DC, Xerri L, Burns CJ. 2020. Discovery of taniborbactam (VNRX-5133): a broad-spectrum serine- and metallo-β-lactamase inhibitor for carbapenem-resistant bacterial infections. J Med Chem 63:2789–2801. doi:10.1021/acs.jmedchem.9b01518. 31765155 PMC7104248

[22] Food and Drug Administration. 2019. FDA news release: Recarbrio: FDA approves new treatment for complicated urinary tract and complicated intra-abdominal infections. Food and Drug Administration, Silver Spring, MD. https://www.fda.gov/news-events/press-announcements/fda-approves-new-treatment-complicated-urinary-tract-and-complicated-intra-abdominal-infections.

[23] Food and Drug Administration. 2017. FDA news release: FDA approves new antibacterial drug. Food and Drug Administration, Silver Spring, MD. https://www.fda.gov/news-events/press-announcements/fda-approves-new-antibacterial-drug.

[24] Food and Drug Administration. 2015. Drug trials snapshot: AVYCAZ (cUTI). Food and Drug Administration, Silver Spring, MD. https://www.fda.gov/drugs/drug-approvals-and-databases/drug-trials-snapshot-avycaz-cuti.

[25] Food and Drug Administration. 2019. FDA news release: FDA approves new treatment for hospital-acquired and ventilator-associated bacterial pneumonia. Food and Drug Administration, Silver Spring, MD. https://www.fda.gov/news-events/press-announcements/fda-approves-new-treatment-hospital-acquired-and-ventilator-associated-bacterial-pneumonia.

[26] Chatwin CL, Hamrick JC, John K, Burns CJ, Xerri L, Moeck G, Pevear DC. 2019. Selection of CTB as the partner antibiotic for the oral β-lactamase inhibitor VNRX-7145. ECCMID poster P1181.

[27] Lin C, Radwanski E, Affrime M, Cayen MN. 1995. Multiple-dose pharmacokinetics of ceftibuten in healthy volunteers. Antimicrob Agents Chemother 39:356–358. doi:10.1128/aac.39.2.356. 7726497 PMC162542

[28] Pernix Therapeutics. 2010. CEDAX package insert. Pernix Therapeutics, Gonzales, LA. https://www.accessdata.fda.gov/drugsatfda_docs/label/2011/050686s016lbl.pdf.

[29] Shields RK, Chen L, Cheng S, Chavda KD, Press EG, Snyder A, Pandey R, Doi Y, Kreiswirth BN, Nguyen MH, Clancy CJ. 2017. Emergence of ceftazidime-avibactam resistance due to plasmid-borne *bla*_KPC-3_ mutations during treatment of carbapenem-resistant Klebsiella pneumoniae infections. Antimicrob Agents Chemother 61:e02097-16. doi:10.1128/AAC.02097-16. 28031201 PMC5328542

[30] Clinical and Laboratory Standards Institute. 2018. Methods for dilution antimicrobial susceptibility tests for bacteria that grow aerobically, approved standard, 11th ed. CLSI document M07-Ed11. CLSI, Wayne, PA.

[31] Clinical and Laboratory Standards Institute. 2020. Performance standards for antimicrobial susceptibility testing, 30th ed. CLSI document M100. CLSI, Wayne, PA.

[32] Dressel D, Hackel M, Sahm D. 2019. Impact of variations in susceptibility testing parameters on the in vitro activity of ceftibuten in combination with VNRX-7145. ASM Microbe poster CPHM-860.

[33] Weiss WJ, Petersen PJ, Murphy TM, Tardio L, Yang Y, Bradford PA, Venkatesan AM, Abe T, Isoda T, Mihira A, Ushirogochi H, Takasake T, Projan S, O’Connell J, Mansour TS. 2004. In vitro and in vivo activities of novel 6-methylidene penems as beta-lactamase inhibitors. AAC 48:4589–4596. doi:10.1128/AAC.48.12.4589-4596.2004. PMC529194 15561830

[34] Eurofins. 2021. Specialized panels. https://www.eurofinsdiscoveryservices.com/services/safety-and-efficacy/safety-pharmacology/specialized-panels/.

[35] ClinicalTrials.gov. 2020. Sulopenem versus ertapenem for complicated intra-abdominal infection (cIAI). https://www.clinicaltrials.gov/ct2/show/NCT03358576?term=sulopenemdraw=2rank=4.

[36] Iterum Therapeutics. 2019. Iterum Therapeutics announces topline results from phase 3 clinical trial of oral and IV sulopenem for the treatment of complicated intra-abdominal infections. https://www.iterumtx.com/news/press-releases/detail/29/iterum-therapeutics-announces-topline-results-from-phase-3.

[37] ClinicalTrials.gov. 2020. Sulopenem followed by sulopenem-etzadroxil/probenecid vs ertapenem followed by Cipro for complicated UTI in adults. https://www.clinicaltrials.gov/ct2/show/NCT03357614?term=sulopenem&draw=2&rank=3.

[38] Spero Therapeutics. 2021. Tebipenem HBr: oral carbapenem in development for treatment of complicated urinary tract infections. https://sperotherapeutics.com/pipeline/tebipenem-hbr-oral-gram-negative-program/.

[39] Karaiskos I, Giamarellou H. 2020. Carbapenem-sparing strategies for ESBL producers: when and how. Antibiotics (Basel) 9:61. doi:10.3390/antibiotics9020061. PMC7167803 32033322

[40] Viale P, Giannella M, Bartoletti M, Tedeschi S, Lewis R. 2015. Considerations about antimicrobial stewardship in settings with epidemic extended-spectrum β-lactamase-producing or carbapenem-resistant Enterobacteriaceae. Infect Dis Ther 4:65–83. doi:10.1007/s40121-015-0081-y. 26362292 PMC4569644

[41] Gutiérrez-Gutiérrez B, Rodríguez-Baño J. 2019. Current options for the treatment of infections due to extended-spectrum beta-lactamase-producing Enterobacteriaceae in different groups of patients. Clin Microbiol Infect 25:932–942. doi:10.1016/j.cmi.2019.03.030. 30986558

[42] Rodríguez-Baño J, Gutiérrez-Gutiérrez B, Machuca I, Pascual A. 2018. Treatment of infections caused by extended-spectrum-beta-lactamase-, AmpC-, and carbapenemase-producing Enterobacteriaceae. Clin Microbiol Rev 31:e00079-17. doi:10.1128/CMR.00079-17. 29444952 PMC5967687

[43] Aslan AT, Akova M. 2019. Extended spectrum β-lactamase producing enterobacteriaceae: carbapenem sparing options. Expert Rev Anti Infect Ther 17:969–981. doi:10.1080/14787210.2019.1693258. 31722185

[44] Hecker SJ, Reddy KR, Lomovskaya O, Griffith DC, Rubio-Aparicio D, Nelson K, Tsivkovski R, Sun D, Sabet M, Tarazi Z, Parkinson J, Totrov M, Boyer SH, Glinka TW, Pemberton OA, Chen Y, Dudley MN. 2020. Discovery of cyclic boronic acid QPX7728, an ultrabroad-spectrum inhibitor of serine and metallo-β-lactamases. J Med Chem 63:7491–7507. doi:10.1021/acs.jmedchem.9b01976. 32150407

[45] Durand-Réville TF, Comita-Prevoir J, Zhang J, Wu X, May-Dracka TL, Romero JAC, Wu F, Chen A, Shapiro AB, Carter NM, McLeod SM, Giacobbe RA, Verheijen JC, Lahiri SD, Sacco MD, Chen Y, O'Donnell JP, Miller AA, Mueller JP, Tommasi RA. 2020. Discovery of an orally available diazabicyclooctane inhibitor (ETX0282) of class A, C, and D serine β-lactamases. J Med Chem 63:12511–12525. doi:10.1021/acs.jmedchem.0c00579. 32658473 PMC7927146

[46] Tzouvelekis LS, Markogiannakis A, Psichogiou M, Tassios PT, Daikos GL. 2012. Carbapenemases in Klebsiella pneumoniae and other Enterobacteriaceae: an evolving crisis of global dimensions. Clin Microbiol Rev 25:682–707. doi:10.1128/CMR.05035-11. 23034326 PMC3485753

[47] Munoz-Price LS, Poirel L, Bonomo RA, Schwaber MJ, Daikos GL, Cormican M, Cornaglia G, Garau J, Gniadkowski M, Hayden MK, Kumarasamy K, Livermore DM, Maya JJ, Nordmann P, Patel JB, Paterson DL, Pitout J, Villegas MV, Wang H, Woodford N, Quinn JP. 2013. Clinical epidemiology of the global expansion of Klebsiella pneumoniae carbapenemases. Lancet Infect Dis 13:785–796. doi:10.1016/S1473-3099(13)70190-7. 23969216 PMC4673667

[48] Doi Y, Iovleva A, Bonomo RA. 2017. The ecology of extended-spectrum β-lactamases (ESBLs) in the developed world. J Travel Med 24(suppl_1):S44–S51. doi:10.1093/jtm/taw102. 28521000 PMC5731446

[49] WHO. 2017. WHO publishes list of bacteria for which new antibiotics are urgently needed. https://www.who.int/news/item/27-02-2017-who-publishes-list-of-bacteria-for-which-new-antibiotics-are-urgently-needed.

[50] Yigit H, Queenan AM, Anderson GJ, Domenech-Sanchez A, Biddle JW, Steward CD, Alberti S, Bush K, Tenover FC. 2001. Novel carbapenem-hydrolyzing beta-lactamase, KPC-1, from a carbapenem-resistant strain of Klebsiella pneumoniae. Antimicrob Agents Chemother 45:1151-61. doi:10.1128/AAC.45.4.1151-1161.2001. 11257029 PMC90438

[51] Nordmann P, Poirel L. 2014. The difficult-to-control spread of carbapenemase producers among Enterobacteriaceae worldwide. Clin Microbiol Infect 20:821–830. doi:10.1111/1469-0691.12719. 24930781

[52] Mairi A, Pantel A, Sotto A, Lavigne JP, Touati A. 2018. OXA-48-like carbapenemases producing Enterobacteriaceae in different niches. Eur J Clin Microbiol Infect Dis 37:587–604. doi:10.1007/s10096-017-3112-7. 28990132

[53] Pitout JDD, Peirano G, Kock MM, Strydom KA, Matsumura Y. 2019. The global ascendency of OXA-48-type carbapenemases. Clin Microbiol Rev 33:e00102-19. doi:10.1128/CMR.00102-19. 31722889 PMC6860007

[54] Abdelraouf K, Stainton SM, Nicolau DP. 2019. *In vivo* pharmacodynamic profile of ceftibuten-clavulanate combination against extended-spectrum-β-lactamase-producing Enterobacteriaceae in the murine thigh infection model. Antimicrob Agents Chemother 63:e00145-19. doi:10.1128/AAC.00145-19. 31061165 PMC6591625

[55] ClinicalTrials.gov. 2021. VNRX-5024 safety and PK in healthy adult volunteers. https://www.clinicaltrials.gov/ct2/show/NCT04314206?term=venatorx&draw=2&rank=3.

[56] Pevear DC, Trout RE, McLaughlin L, Hamrick JC, Moeck G. 2019. Oral bioavailability of novel β-lactamase inhibitor VNRX-7145 in rats, dogs and non-human primates. ASM Microbe poster AAR-728.

[57] Morinaka A, Tsutsumi Y, Yamada M, Suzuki K, Watanabe T, Abe T, Furuuchi T, Inamura S, Sakamaki Y, Mitsuhashi N, Ida T, Livermore DM. 2015. OP0595, a new diazabicyclooctane: mode of action as a serine β-lactamase inhibitor, antibiotic and β-lactam ‘enhancer’. J Antimicrob Chemother 70:2779–2786. doi:10.1093/jac/dkv166. 26089439

[58] Rajavel M, Kumar V, Nguyen H, Wyatt J, Marshall SH, Papp-Wallace KM, Deshpande P, Bhavsar S, Yeole R, Bhagwat S, Patel M, Bonomo RA, van den Akker F. 2021. Structural characterization of diazabicyclooctane β-lactam “enhancers” in complex with penicillin-binding proteins PBP2 and PBP3 of Pseudomonas aeruginosa. mBio 12:e03058-20. doi:10.1128/mBio.03058-20. 33593978 PMC8545096

[59] Mushtaq S, Vickers A, Woodford N, Haldimann A, Livermore DM. 2019. Activity of nacubactam (RG6080/OP0595) combinations against MBL-producing Enterobacteriaceae. J Antimicrob Chemother 74:953–960. doi:10.1093/jac/dky522. 30590470

[60] Morinaka A, Tsutsumi Y, Yamada K, Takayama Y, Sakakibara S, Takata T, Abe T, Furuuchi T, Inamura S, Sakamaki Y, Tsujii N, Ida T. 2016. In vitro and in vivo activities of OP0595, a new diazabicyclooctane, against CTX-M-15-positive Escherichia coli and KPC-positive Klebsiella pneumoniae. Antimicrob Agents Chemother 60:3001–3006. doi:10.1128/AAC.02704-15. 26953205 PMC4862534

[61] Livermore DM, Mushtaq S, Warner M, Vickers A, Woodford N. 2017. In vitro activity of cefepime/zidebactam (WCK 5222) against Gram-negative bacteria. J Antimicrob Chemother 72:1373–1385. doi:10.1093/jac/dkw593. 28158732

[62] Livermore DM, Mushtaq S, Warner M, Woodford N. 2015. Activity of OP0595/β-lactam combinations against Gram-negative bacteria with extended-spectrum, AmpC and carbapenem-hydrolysing β-lactamases. J Antimicrob Chemother 70:3032–3041. doi:10.1093/jac/dkv239. 26311835

[63] Moya B, Barcelo IM, Bhagwat S, Patel M, Bou G, Papp-Wallace KM, Bonomo RA, Oliver A. 2017. WCK 5107 (Zidebactam) and WCK 5153 are novel inhibitors of PBP2 showing potent “β-lactam enhancer” activity against Pseudomonas aeruginosa, including multidrug-resistant metallo-β-lactamase-producing high-risk clones. Antimicrob Agents Chemother 61:e02529-16. doi:10.1128/AAC.02529-16. 28289035 PMC5444176

[64] Avery LM, Abdelraouf K, Nicolau DP. 2019. Assessment of the in vivo pharmacodynamic profile of ceftibuten (CTB)/VNRX-7145 combination against serine β-lactamase-producing Enterobacteriaceae (SBL-EB) in the neutropenic murine thigh infection model. ASM Microbe poster AAR-727. 10.1128/AAC.00145-19 PMC6591625 31061165

[65] Pulse ME, Weiss WJ, Nguyen P, Valtierre D, Peterson K, Carter K, Weiss J, Deviney A, Elmquist G, Moeck G, Trout RE, Hamrick J, Pevear DC. 2019. Efficacy of ceftibuten + VNRX-7145, a novel β-lactamase inhibitor, against KPC-2 and ESBL E. coli strains in a murine UTI model. ASM Microbe poster AAR-726.

[66] Avery LM, Abdelraouf K, Nicolau DP. In vivo pharmacodynamics of VNRX-7145 in the neutropenic murine thigh infection model when administered in combination with humanized exposures of twice daily ceftibuten against serine-β-lactamase-producing Enterobacteriaceae. IDWeek 20 19 poster 682.

[67] Castanheira M, Deshpande LM, Mendes RE, Canton R, Sader HS, Jones RN. 2019. Variations in the occurrence of resistance phenotypes and carbapenemase genes among *Enterobacteriaceae* isolates in 20 years of the SENTRY antimicrobial surveillance program. Open Forum Infect Dis 6:S23–S33. doi:10.1093/ofid/ofy347. 30895212 PMC6419900

[68] Hackel M, Sahm D. 2019. In vitro activity of ceftibuten in combination with VNRX-7145 and comparators against 1,066 UTI isolates non-susceptible to amoxicillin-clavulanate and levofloxacin. ASM Microbe poster AAR-721.

[69] Golemi D, Maveyraud L, Vakulenko S, Samama JP, Mobashery S. 2001. Critical involvement of a carbamylated lysine in catalytic function of class D β-lactamases. Proc Natl Acad Sci U S A 98:1420–1428. doi:10.1073/pnas.241442898. PMC64673 11724923

[70] Clinical Laboratory Standards Institute. 1999. Methods for determining bactericidal activity of antimicrobial agents, approved guidelines. CLSI document M26-A. CLSI, Wayne, PA.

