# Supplementary material for: Microbiological Characterization of VNRX-5236, a Broad-Spectrum β-Lactamase Inhibitor for Rescue of the Orally Bioavailable Cephalosporin Ceftibuten as a Carbapenem-Sparing Agent against Strains of Enterobacterales Expressing Extended-Spectrum β-Lactamases and Serine Carbapenemases
Source: Antimicrob Agents Chemother. 2021 Jul 16;65(8):e00552-21. doi: 10.1128/AAC.00552-21 (PMC8284453; doi:10.1128/AAC.00552-21)
Supplement: Supplemental file 1 — Supplemental Tables S1 to S9. Download AAC00552-21_Supp_1_seq1.pdf, PDF file, 0.3 MB [file aac00552-21_supp_1_seq1.pdf]

Supplemental Data

Table S1. Absence of stand-alone antibacterial activity of VNRX-5236 and VNRX-7145.

Strain	*ID*	*Phenotype*	*MIC (µg/mL)*		
Gram Positive			VNRX-5236	VNRX-7145	Ceftazidime
S. aureus	29213	MSSA	≥128	≥128	8
S. aureus	33591	MRSA	≥128	≥128	128
E. faecium	134529	VRE	≥128	≥128	≥128
E. faecalis	29212	WT	≥128	≥128	≥128
Gram Negative					
E. coli	25922	WT	≥128	≥128	0.25
E. coli	J53	SHV-5, AmpC, TEM-1	≥128	≥128	≥128
E. coli	DH5α	TEM-1	≥128	≥128	4
E. coli	DH5α	CTX-M-15	≥128	≥128	32
K. pneumoniae	CI09	WT	≥128	≥128	0.25
K. pneumoniae	UMM	SHV-5, KPC-2, TEM-1	≥128	≥128	16
E. coli	AG100	Efflux (+)	≥128	≥128	0.5
E. coli	AG100 A	Efflux (-)	≥128	≥128	0.25
P. aeruginosa	PAO1	WT	≥128	≥128	2

Legend: All testing conducted according to CLSI broth microdilution method. Abbreviations: MSSA, methicillin-susceptible *Staphylococcus aureus*; MRSA, methicillin-resistant *S. aureus*; VRE, vancomycin-resistant enterococcus; Efflux(-), AcrAB efflux system knockout.

Table S2. Partner selection: Antibacterial activity of partner oral β-lactam against ESBL-expressing Enterobacterales with VNRX-5236 fixed at 4 μg/mL.

VNRX-5236 +		0.03	0.06	0.12	0.25	0.5	1	2	4	8	16	32	≥64
Ceftibuten	N at MIC	1	8	12	**3**	0	1	0	0	0	0	0	0
	Cumulative %	4	36	84	96	96	**100**	100	100	100	100	100	100
Amoxicillin	N at MIC	0	0	0	0	0	0	0	0	0	3	4	**18**
	Cumulative %	0	0	0	0	0	0	0	0	0	12	28	**100**
Cefaclor	N at MIC	0	0	0	0	5	11	**8**	0	0	1	0	0
	Cumulative %	0	0	0	0	20	64	96	96	96	100	**100**	100
Cefdinir	N at MIC	0	0	5	7	8	4	1	0	0	0	0	0
	Cumulative %	0	0	20	48	80	96	**100**	100	100	100	100	100
Cefditoren	N at MIC	0	0	4	6	8	6	1	0	0	0	0	0
	Cumulative %	0	0	16	40	72	96	**100**	100	100	100	100	100
Cefixime	N at MIC	0	0	4	8	7	3	3	0	0	0	0	0
	Cumulative %	0	0	16	48	76	88	100	**100**	100	100	100	100
Cefpodoxime	N at MIC	0	0	2	3	7	8	5	0	0	0	0	0
	Cumulative %	0	0	8	20	48	80	100	100	**100**	100	100	100
Cefuroxime	N at MIC	0	0	0	0	0	0	3	7	10	5	0	0
	Cumulative %	0	0	0	0	0	0	12	40	80	100	100	**100**
Cephalexin	N at MIC	0	0	0	0	0	0	0	8	15	1	0	1
	Cumulative %	0	0	0	0	0	0	0	32	92	96	96	**100**

Legend: all testing conducted according to CLSI broth microdilution method with VNRX-5236 fixed at 4 μg/mL and β-lactam partner titrated. Results represent modal values of 4 or 5 MIC tests. N=25 strains for each combination tested. MIC_{90} highlighted and boxed.

Table S3. Partner selection: Antibacterial activity of partner oral β-lactam against KPC-expressing Enterobacterales with VNRX-5236 fixed at 4 µg/mL.

		MIC (µg/mL)										
VNRX-5236 +		0.06	0.12	0.25	0.5	1	2	4	8	16	32	≥64
Ceftibuten	N at MIC	6	9	2	4	**4**	0	0	0	0	0	0
	Cumulative %	24	60	68	84	**100**	100	100	100	100	100	100
Amoxicillin	N at MIC	0	0	0	0	0	0	0	0	2	4	**19**
	Cumulative %	0	0	0	0	0	0	0	0	8	24	**100**
Cefaclor	N at MIC	0	0	0	1	3	2	8	3	2	0	**6**
	Cumulative %	0	0	0	4	16	24	56	68	76	76	**100**
Cefdinir	N at MIC	0	3	5	6	2	2	1	2	**3**	0	1
	Cumulative %	0	12	32	56	64	72	76	84	**96**	96	100
Cefditoren	N at MIC	0	1	5	9	4	1	**5**	0	0	0	0
	Cumulative %	0	4	24	60	76	80	**100**	100	100	100	100
Cefixime	N at MIC	0	5	1	10	3	**4**	2	0	0	0	0
	Cumulative %	0	20	24	64	76	**92**	100	100	100	100	100
Cefpodoxime	N at MIC	0	2	5	4	6	2	1	**4**	0	1	0
	Cumulative %	0	8	28	44	68	76	80	**96**	96	100	100
Cefuroxime	N at MIC	0	0	0	0	0	1	2	8	6	2	**6**
	Cumulative %	0	0	0	0	0	4	12	44	68	76	**100**
Cephalexin	N at MIC	0	0	0	0	0	0	3	1	11	2	**8**
	Cumulative %	0	0	0	0	0	0	12	16	60	68	**100**

Legend: all testing conducted according to CLSI broth microdilution method with VNRX-5236 fixed at 4 µg/mL and β-lactam partner titrated. Results represent modal values of 4 or 5 MIC tests. N=25 strains for each combination tested. MIC_{90} highlighted and boxed.

Table S4. Partner selection: Antibacterial activity of partner oral β-lactam against Ambler class C-expressing Enterobacterales with VNRX-5236 fixed at 4 µg/mL.

		MIC (µg/mL)											
VNRX-5236 +		0.03	0.06	0.12	0.25	0.5	1	2	4	8	16	32	≥64
Ceftibuten	N at MIC	1	5	7	4	4	3	1	0	0	0	0	0
	Cumulative %	4	24	52	68	84	**96**	100	100	100	100	100	100
Amoxicillin	N at MIC	0	0	0	0	0	0	1	1	1	2	3	17
	Cumulative %	0	0	0	0	0	0	4	8	12	20	32	**100**
Cefaclor	N at MIC	0	0	0	0	3	7	6	4	1	1	**2**	1
	Cumulative %	0	0	0	0	12	40	64	80	84	88	**96**	100
Cefdinir	N at MIC	0	1	2	10	5	2	**3**	1	0	1	0	0
	Cumulative %	0	4	12	52	72	80	**92**	96	96	100	100	100
Cefditoren	N at MIC	0	1	4	5	7	5	**2**	1	0	0	0	0
	Cumulative %	0	4	20	40	68	88	**96**	100	100	100	100	100
Cefixime	N at MIC	0	1	3	5	5	4	2	**4**	1	0	0	0
	Cumulative %	0	4	16	36	56	72	80	**96**	100	100	100	100
Cefpodoxime	N at MIC	0	0	1	4	8	5	3	1	**2**	1	0	0
	Cumulative %	0	0	4	20	52	72	84	88	**96**	100	100	100
Cefuroxime	N at MIC	0	0	0	0	0	0	2	4	8	5	3	**3**
	Cumulative %	0	0	0	0	0	0	8	24	56	76	88	**100**
Cephalexin	N at MIC	0	0	0	0	0	0	0	6	9	4	2	**4**
	Cumulative %	0	0	0	0	0	0	0	24	60	76	84	**100**

Legend: all testing conducted according to CLSI broth microdilution method with VNRX-5236 fixed at 4 µg/mL and β-lactam partner titrated. Results represent modal values of 4 or 5 MIC tests. N=25 strains for each combination tested. MIC_{90} highlighted and boxed.

Table S5. Partner selection: Antibacterial activity of partner oral β-lactam against OXA-expressing Enterobacterales with VNRX-5236 fixed at 4 μg/mL.

		MIC (μg/mL)											
VNRX-5236 +		0.03	0.06	0.12	0.25	0.5	1	2	4	8	16	32	≥64
Ceftibuten	N at MIC	1	5	10	4	1	**3**	1	0	0	0	0	0
	Cumulative %	4	24	64	80	84	**96**	100	100	100	100	100	100
Amoxicillin	N at MIC	0	0	0	0	0	0	0	0	0	0	2	**23**
	Cumulative %	0	0	0	0	0	0	0	0	0	0	8	**100**
Cefaclor	N at MIC	0	0	0	0	0	3	5	6	6	1	0	**4**
	Cumulative %	0	0	0	0	0	12	32	56	80	84	84	**100**
Cefdinir	N at MIC	0	0	0	0	1	2	10	2	3	4	**1**	2
	Cumulative %	0	0	0	0	4	12	52	60	72	88	**92**	100
Cefditoren	N at MIC	0	0	0	1	8	9	4	**1**	0	2	0	0
	Cumulative %	0	0	0	4	36	72	88	**92**	92	100	100	100
Cefixime	N at MIC	0	0	2	10	8	1	**2**	0	1	1	0	0
	Cumulative %	0	0	8	48	80	84	**92**	92	96	100	100	100
Cefpodoxime	N at MIC	0	0	0	3	7	8	3	1	**1**	0	1	1
	Cumulative %	0	0	0	12	40	72	84	88	**92**	92	96	100
Cefuroxime	N at MIC	0	0	0	0	0	0	0	2	11	6	2	**4**
	Cumulative %	0	0	0	0	0	0	0	8	52	76	84	**100**
Cephalexin	N at MIC	0	0	0	0	0	0	0	3	9	7	2	**4**
	Cumulative %	0	0	0	0	0	0	0	12	48	76	84	**100**

Legend: all testing conducted according to CLSI broth microdilution method with VNRX-5236 fixed at 4 μg/mL and β-lactam partner titrated. Results represent modal values of 4 or 5 MIC tests. N=25 strains for each combination tested. MIC_{90} highlighted and boxed.

Table S6. Activity of ceftibuten + VNRX-5236 and comparators against Enterobacterales expressing ESBLs.

Species	Strain ID	Enzyme Content	Amoxicillin		Ceftibuten			Sulopenem	Tebipenem
			-	CLA (2:1)	-	VNRX-5236 (4 µg/mL)	CLA (4 µg/mL)		
E. coli	1924	TEM-12	≥256	16	0.5	0.12	0.5	0.5	0.03
E. coli	2150	SHV-3, TEM-1	≥256	8	0.5	0.12	0.5	0.5	0.03
E. coli	2806	SHV-12, TEM-1	≥256	8	1	0.12	0.5	0.5	≤0.016
E. coli	3327	SHV-12, TEM-1	≥256	32	2	0.12	1	1	0.03
K. pneumoniae	115468	TEM-1	≥256	16	2	0.03	0.03	0.03	0.06
K. pneumoniae	153239	SHV-11, TEM-1	≥256	8	0.12	0.06	0.06	0.06	0.03
K. pneumoniae	304487	SHV-12, TEM-1	≥256	8	8	0.25	0.25	0.25	0.03
K. pneumoniae	319478	CTX-M-3, SHV-12, TEM-1	≥256	32	32	0.06	0.12	0.12	0.06
K. pneumoniae	329633	SHV-5, TEM-1	≥256	16	4	0.06	0.03	0.03	0.03
E. coli	ESBL 4	CTX-M-15, TEM-1	≥256	16	16	0.12	0.5	0.5	0.03
E. coli	ESBL 5	CTX-M-15, TEM-1	≥256	32	≥64	0.12	0.5	0.5	0.12
K. pneumoniae	ESBL 7 #1	SHV-12, TEM-1	≥256	8	8	0.12	0.12	0.12	≤0.016
K. pneumoniae	ESBL 7 #2	CTX-M-15, SHV-12, TEM-1	≥64	≥64	≥64	1	≥64	0.25	0.12
K. pneumoniae	ESBL 8 #1	SHV-12, TEM-1	≥256	8	8	0.06	0.03	0.03	≤0.016
K. pneumoniae	ESBL 8 #2	SHV-12, TEM-1	≥64	8	8	0.12	0.03	0.03	0.03
K. pneumoniae	ESBL 10	SHV-12, TEM-1	≥256	8	8	0.25	0.12	0.12	0.03
K. pneumoniae	KI (KC1)	TEM-10	≥64	8	0.25	0.06	0.12	0.06	0.03
K. pneumoniae	KP 3	SHV-1, TEM-1	≥64	≥64	4	0.12	0.12	0.12	0.06
E. coli	SI-LP377	CTX-M-2	≥256	16	1	0.06	0.12	0.12	≤0.016
E. coli	SI-M004	GES-12, SHV-2	≥256	16	≥64	0.12	0.5	0.5	≤0.016
E. coli	SI-NO36	SHV-11, TEM-1	≥256	8	0.25	0.06	0.25	0.25	≤0.016
E. coli	SI-V502	CTX-M-15, TEM-29	≥256	32	0.5	0.25	0.25	0.25	0.06
		Range	≥64-≥256	8-≥64	0.12-≥64	0.03-1	0.03-≥64	0.03-1	≤0.016-0.12
		MIC_{50}	≥256	16	4	0.12	0.12	0.12	0.03
		MIC_{90}	≥256	32	≥64	0.25	0.5	0.5	0.06

Legend: All testing conducted according to CLSI broth microdilution method. Results represent modal values of 4 or 5 MIC tests. Abbreviations: CLA, clavulanic acid.

Table S7. Activity of ceftibuten + VNRX-5236 and comparators against Enterobacterales expressing KPCs.

Species	Strain ID	Enzyme Content	Amoxicillin		Ceftibuten			Sulopenem	Tebipenem
			-	CLA (2:1)	-	VNRX-5236 (4 µg/mL)	CLA (4 µg/mL)		
K. pneumoniae	155140	AmpC, KPC-2, SHV-12, TEM-1	≥256	≥256	≥64	1	≥64	≥64	≥64
E. coli	786978	KPC	≥64	≥64	32	0.5	16	≥64	≥64
K. pneumoniae	845661	KPC	≥64	≥64	32	1	16	≥64	≥64
K. pneumoniae	845662	KPC	≥64	≥64	16	1	16	≥64	≥64
K. pneumoniae	845665	KPC	≥64	≥64	32	0.5	32	≥64	≥64
K. pneumoniae	845667	KPC	≥64	≥64	16	1	32	≥64	≥64
K. pneumoniae	845670	KPC	≥64	32	0.5	0.06	0.5	16	32
K. pneumoniae	845904	KPC	≥64	32	1	0.06	1	16	≥64
K. pneumoniae	847204	KPC	≥64	≥64	16	0.25	16	32	≥64
K. pneumoniae	847375	KPC	≥64	≥64	8	0.12	8	32	≥64
K. pneumoniae	847378	KPC	≥256	128	8	0.12	8	32	≥64
K. pneumoniae	847379	KPC	≥256	64	8	0.06	0.5	8	16
K. pneumoniae	847383	KPC	≥256	128	8	0.12	4	32	≥64
K. pneumoniae	847384	KPC	≥256	≥256	8	0.06	4	≥64	≥64
K. pneumoniae	847387	KPC	≥64	≥64	16	0.12	4	32	≥64
E. cloacae	847426	KPC	≥256	≥256	≥64	0.5	≥64	≥64	≥64
E. cloacae	847429	KPC	≥64	≥64	16	0.5	16	≥64	≥64
K. oxytoca	847537	KPC	≥256	≥256	≥64	0.25	32	≥64	≥64
K. oxytoca	847539	KPC	≥256	64	0.5	0.06	0.25	4	8
K. pneumoniae	847747	KPC	≥256	128	4	0.06	4	32	≥64
K. pneumoniae	848832	KPC	≥256	≥256	16	0.12	8	≥64	≥64
K. pneumoniae	848844	KPC	≥256	≥256	16	0.12	8	32	≥64
K. pneumoniae	897067	KPC	≥256	128	2	0.12	1	8	32
E. cloacae	OIMGH49	p99 AmpC, ACT-2, KPC-2, TEM-1	≥64	≥64	≥64	0.12	≥64	≥64	32
K. pneumoniae	UMM	KPC-2, SHV-5, TEM-1	≥256	64	1	0.12	0.5	16	32
		Range	≥64-≥256	32-≥256	0.5-≥64	0.06-1	0.25-≥64	4-≥64	8-≥64
		MIC_{50}	≥256	≥64	16	0.12	8	32	≥64
		MIC_{90}	≥256	≥256	≥64	1	≥64	≥64	≥64

Legend: All testing conducted according to CLSI broth microdilution method. Results represent modal values of 4 or 5 MIC tests. Abbreviations: CLA, clavulanic acid.

Table S8. Activity of ceftibuten + VNRX-5236 and comparators against Enterobacterales expressing Ambler class C enzymes.

Species	Strain ID	Enzyme Content	Amoxicillin		Ceftibuten			Sulopenem	Tebipenem
			-	CLA (2:1)	-	VNRX-5236 (4 µg/mL)	CLA (4 µg/mL)		
E. coli	128	AmpC, SHV-12, TEM-1, Toho-2	128	8	2	0.06	0.12	0.03	≤0.016
E. coli	3102	AmpC, CTX-M-15, TEM-1	32	32	32	0.25	32	0.03	≤0.016
E. aerogenes	110099	AmpC, SHV-12	≥256	128	32	0.5	32	0.06	0.12
E. aerogenes	111707	AmpC, TEM-1	≥256	16	2	0.12	1	0.12	0.03
E. aerogenes	111902	AmpC, TEM-1	≥256	64	≥64	1	≥64	0.25	0.12
K. pneumoniae	129032	MOX-2, SHV-12, TEM-1	≥256	32	16	0.12	0.06	0.06	0.03
S. marcescens	132253	AmpC, CTX-M-3, OXA-1, SHV-12, TEM-1	≥256	≥256	≥64	0.5	4	2	0.5
C. freundii	134390	AmpC, TEM-1	≥256	16	4	0.03	1	0.06	0.03
S. marcescens	134434	AmpC, SHV-12, TEM-1	≥256	128	32	1	8	8	0.5
S. marcescens	142397	AmpC, SHV-7, TEM-1	≥256	≥256	≥64	2	≥64	2	0.25
S. marcescens	156212	AmpC, SHV-12, TEM-1	≥256	≥256	8	0.12	4	1	0.25
K. pneumoniae	178279	AmpC, SHV-12, TEM-1	≥256	16	8	0.06	0.06	0.06	0.03
S. marcescens	192478	AmpC, SHV-12, TEM-1	≥256	128	8	0.12	0.25	0.25	0.12
K. pneumoniae	212027	AmpC, FOX-5, TEM-1	≥256	32	16	0.06	16	0.03	0.03
K. pneumoniae	217917	CMY-2, TEM-1	≥256	32	8	0.12	0.06	0.06	0.06
S. marcescens	218068	AmpC, SHV-7, TEM-1	≥256	128	32	0.25	1	4	0.25
K. pneumoniae	301313	AmpC, SHV-12, TEM-1	≥256	32	≥64	0.25	0.12	0.12	0.06
K. pneumoniae	398845	CMY-2, SHV-12, TEM-1	≥256	32	16	0.12	0.06	0.06	0.03
E. coli	CH1	AmpC, CTX-M-15, SHV-1, SHV-5, TEM-1	≥256	64	≥64	0.5	≥64	0.06	0.06
E. coli	EC469	AmpC, SHV-12, TEM-1	128	8	2	0.06	0.12	0.03	≤0.016
E. coli	J53	AmpC, SHV-5, TEM-1	128	16	8	0.5	2	0.06	≤0.016
K. pneumoniae	SI- F100	CMY-2, SHV-5, TEM-1	≥256	8	2	0.06	0.06	0.03	≤0.016
P. mirabilis	SI-F014	AmpC, TEM-72	≥256	16	≥64	1	2	1	2
E. coli	SI-P026TC	CMY-2, TEM-1	≥256	64	≥64	0.25	≥64	0.25	0.12
E. coli	SI-PO26	AmpC, CMY-2, TEM-1	≥256	64	≥64	0.12	≥64	0.12	0.03
		Range	32-≥256	8-≥256	2-≥64	0.03-2	0.06-≥64	0.03-8	≤0.016-2
		MIC_{50}	≥256	32	16	0.12	2	0.06	0.06
		MIC_{90}	≥256	≥256	≥64	1	≥64	2	0.5

Legend: All testing conducted according to CLSI broth microdilution method. Results represent modal values of 4 or 5 MIC tests. Abbreviations: CLA, clavulanic acid.

Table S9. Activity of ceftibuten + VNRX-5236 and comparators against Enterobacterales expressing Oxacillinases.

Species	Strain ID	Enzyme Content	Amoxicillin		Ceftibuten			Sulopenem	Tebipenem
			-	CLA (2:1)	-	VNRX-5236 (4 µg/mL)	CLA (4 µg/mL)		
K. pneumoniae	6299	OXA-163	≥256	≥256	8	0.12	4	1	4
K. pneumoniae	515746	OXA-48	≥256	≥256	16	0.03	0.12	2	1
K. pneumoniae	515746	OXA-48	≥256	≥256	16	0.06	0.25	4	0.5
K. pneumoniae	664438	OXA-48	≥256	≥256	16	0.12	2	8	8
K. pneumoniae	664439	OXA-48	≥256	≥256	16	0.12	1	8	4
E. coli	664507	OXA-48	≥256	≥256	16	0.12	4	8	4
K. pneumoniae	664509	OXA-48	≥256	≥256	≥64	1	32	≥64	≥64
E. coli	664516	OXA-48	≥256	≥256	32	0.5	8	16	8
K. pneumoniae	664518	OXA-48	≥256	≥256	32	0.25	2	8	4
E. coli	664520	OXA-48	≥256	≥256	16	0.12	4	8	4
E. coli	664522	OXA-48	≥256	≥256	32	0.25	8	16	8
E. coli	664523	OXA-48	≥256	≥256	16	0.12	4	4	4
E. coli	664524	OXA-48	≥256	≥256	16	0.12	4	8	4
E. coli	664525	OXA-48	≥256	≥256	32	0.25	16	16	8
E. coli	664526	OXA-48	≥256	≥256	16	0.12	4	8	4
E. coli	664528	OXA-48	≥256	≥256	16	0.12	4	8	4
K. pneumoniae	722870	OXA-48	≥256	≥256	8	0.06	1	8	1
E. coli	787112	OXA-48	≥256	≥256	≥64	1	16	16	8
K. pneumoniae	BAH	OXA-204	≥256	≥256	≥64	0.25	≥64	4	0.5
K. pneumoniae	DEL	OXA-232	≥256	≥256	≥64	2	32	32	≥64
K. pneumoniae	DOV	OXA-48	≥256	128	8	0.06	0.06	2	0.5
K. pneumoniae	KIRK	OXA-162	≥256	≥256	8	0.06	0.5	4	1
E. coli	SI-422	OXA-48	≥256	≥256	0.25	0.12	0.25	4	1
K. pneumoniae	SI-C17	OXA-48	≥256	≥256	≥64	1	8	16	16
E. coli	VER	OXA-48	≥256	≥256	0.06	0.06	0.12	4	1
		Range	≥256	128-≥256	0.06-≥64	0.03-2	0.06-≥64	1-≥64	0.5-≥64
		MIC_{50}	≥256	≥256	16	0.12	4	8	4
		MIC_{90}	≥256	≥256	≥64	1	32	16	16

Legend: All testing conducted according to CLSI broth microdilution method. Results represent modal values of 4 or 5 MIC tests. Abbreviations: CLA, clavulanic acid.
